# What does it mean to be an agent?

**DOI:** 10.3389/fpsyg.2023.1273470

**Published:** 2023-10-17

**Authors:** Meshandren Naidoo

**Affiliations:** School of Law, University of KwaZulu-Natal, Durban, South Africa

**Keywords:** agency, artificial intelligence, autonomy, explanations, personhood, semantics, complex system, mechanics and dynamics

## Abstract

Artificial intelligence (AI) has posed numerous legal–ethical challenges. These challenges are particularly acute when dealing with AI demonstrating substantial computational prowess, which is then correlated with agency or autonomy. A common response to considering this issue is to inquire whether an AI system is “conscious” or not. If it is, then it could constitute an agent, actor, or person. This framing is, however, unhelpful since there are many unresolved questions about consciousness. Instead, a practical approach is proposed, which could be used to better regulate new AI technologies. The value of the practical approach in this study is that it (1) provides an empirically observable, testable framework that contains predictive value; (2) is derived from a data-science framework that uses semantic information as a marker; (3) relies on a self-referential logic which is fundamental to agency; (4) enables the “grading” or “ranking” of AI systems, which provides an alternative method (as opposed to current risk-tiering approaches) and measure to determine the suitability of an AI system within a specific domain (e.g., such as social domains or emotional domains); (5) presents consistent, coherent, and higher informational content as opposed to other approaches; (6) fits within the conception of what informational content “laws” are to contain and maintain; and (7) presents a viable methodology to obtain “agency”, “agent”, and “personhood”, which is robust to current and future developments in AI technologies and society.

## 1. Introduction and limitations

This paper aimed to establish a robust account of agency which can be applied to many kinds of systems, including AI systems. This raises further sub-questions, such as (1) what does it mean to be an agent; and (2) what markers are there to determine an agent? An account of the agency must provide answers to those questions in a generally determinable manner. To build an explanatory account of agency, this study evaluates and uses the various logic underpinning “explanations” using the ecological framing of biological organisms as agents of their own evolution. In this light, information-centric quantification tools such as statistical mechanics and bioinformatics would be attractive sources for creating such an account. An important question would be “what is an AI system?” This question is beyond the scope of this article but will be examined in future research. An additional limit is that this methodology describes an empirically testable account of agency, but it will not describe in detail its preferability compared to existing approaches. It is assumed that the reader is familiar with existing approaches.

Evolution is an ecological phenomenon arising from the purposive engagement of organisms with their conditions of existence. It is incorrect to separate evolutionary biology into processes of inheritance, development, selection, and mutation. Instead, the component processes of evolution are jointly caused by the organismal agency and their ecological relations with their affordances. Purposive action is understood to be agents that use features in their environments as affordances that are conducive to their goals. Furthermore, a Kantian approach (see Part B of Supplementary material) is used. It focuses on accounts of agency and personhood as being the intrinsic purposiveness of the agent/person. A Kantian approach is preferable since it is the common framing for many legal constitutions and is a dominant framing mechanism for questions of this kind. Thus, this research moves away from the erroneous “intentional” approach (Sapolsky, 2017).

## 2. The nature of explanations and understanding

### 2.1. Theories and mental models

Explanations usually contain more than theories, in that they involve different bodies of knowledge (Keil, 2006). Explanations create trajectories and lead to understanding among people. They also tend to be more robust than theories. Explanations differ from mental models, which rather speak to formal representations of logical patterns to image-like representations of the works of systems. Mental models are often understood in spatial terms and explanations are not the same as mental modeling. Explanations involve interpretations. The value of explanations in growing knowledge lies in their transactional status and their interpretation (Keil, 2006). Related to this is the question “what does it mean to understand?” When people are probed about their beliefs about the world, coherence often evaporates. Often only fragments about the workings of systems are known, and of these known fragments very few are coherent (Keil, 2006). People's beliefs also tend to contradict one another. They are ignored only until the time when they are made explicit or are pointed out by someone else. This may be because of the limits of working memory. Therefore, not all elements can be considered together at the same time, which would help identify inconsistencies.

### 2.2. Synchronicity and the nature of oscillation

The question that has plagued humans for a long time is how do we come to an agreement on anything? In language, how do we agree on the meaning of words? In behavioral sciences, it is asked how do we know behaviors? In physics, we ask how entangled particles know what the others are doing? Weiner (1948) published *Cybernetics: Or Control and Communication in the Animal and the Machine*. He discussed the problems of communication and control in systems. He used the example of crickets and how they can synchronize their behavior so that their chirps can follow the progression that it does.

The answer is in oscillations or spin; we can observe this in neurons and non-living things such as pendulums synchronizing with each other, which Christiaan Huygens wrote about in 1665 (Redish, 2019). Mathematics then captured the essence of synchronization. There are populations/families of oscillators. Oscillators are things that repeat themselves. A pendulum, for example, is a mechanical oscillator, and a neuron firing in the brain is a cellular oscillator. Birds moving in unison, flying together, are animal oscillators.

#### 2.2.1. Coupling

What is next needed is a coupling mechanism between individuals in a population. Coupling (Stankovski et al., 2015) depends on the population of concern. For neurons, it is the connections between each of them. For animals, it is sight or sound. For particles, it is spin. You can then capture frequency/pulse. There are also weak and strong couplings. Strong couplings mean that there is a stronger statistical tendency for the oscillation relationship/synchronization to take place. For coupling to take place, either strong or weak, there must be a relatively similar innate frequency between individuals, and they must be local (generally). Many different interlinking oscillators apply to humans and other creatures. The Yoshiko Kuramoto mathematical model (Strogatz, 2000) can explain complicated behaviors in complex systems, including perhaps even semantic information. Oscillation and coupling are key components of understanding (perhaps *the key* components). These components explain not only understanding but also relationality and non-verbal/verbal social communication.

#### 2.2.2. The brain

Robert Moore, Victor Eichler, Frederich Stephan, and Irving Zucker discovered the brain regions responsible for governing circadian rhythms. The key structure is the suprachiasmatic nucleus (SCN), which processes information about light and darkness from the retinas. Damaged SCNs impair animal rhythms. Oscillators are the tools used to interlink and relate to others like us. They define what constitutes an “us”. Examples of coupling mechanisms include things such as heat, shape, direction, and vision (eyes, in particular, are a gateway for bonding) (Cornell University, 2022). Previously, the postulation was that mirror neurons enabled us to mimic the behaviors of others in our social group and thus coordinate social or group learning; however, this has not been confirmed (Dickerson et al., 2017). Oscillators and coupling are the modalities of world-building and social organization or communication.

More generally, there are other instances of “understanding” or knowing. These instances involve embodied ontogenetic knowledge: of time, place, circumstance, culture, bodily knowledge (such as sensory information), and the like. For John Vervaeke, this is the four modalities of knowing: (1) participatory knowing; (2) perspectival knowing; (3) procedural knowing; and (4) propositional knowing (Raninen, 2023). Therefore, notions such as “understanding” or “knowing” are not related to thought or mental representations but rather to natural and mechanical processes of relation. This enables a reframing of these concepts such that they do not need to be intimately linked to purely human mental representations.

### 2.3. Patterns, stances, domains, and social/emotion

We can distinguish different explanations by the causal patterns they employ, the stances they invoke, the domains of phenomena they explain, or whether they are value- or emotion-laden (Keil, 2006). Each of these has different trajectories and properties.

#### 2.3.1. Causal patterns

The most common causal relations to which explanations refer are (1) common cause, (2) common effect, (3) linear causal chains, and (4) causal homeostasis (Keil, 2006). Common cause explanations cite a single cause as having a branching set of consequences. These are usually diagnosis-type explanations (such as a bacterial infection that causes many symptoms or a computer virus). Common effect refers to instances where causes converge to create an event. These are common in historical narratives where several causes are attributed to converge and create an event. Linear chains, on the other hand, are degenerate cases of common cause and effect. With these, there is a unique serial chain from a single initial cause to a series of steps through a single effect (Keil, 2006). Causal homeostatic explanations are fundamental to natural kinds of explanations. These explain why sets of things endure as stable sets of properties. This type does not explain how a cause progresses over time to create effect(s), but rather how an interlocking set of causes and effects results in a set of properties that endure in combination over time as a stable set. This stable set is then of a natural kind. Some explanations are easier to follow, while others are more difficult and hence “unnatural”. Furthermore, some explanations are often understood to be domain-based, although this is not necessarily the case (Keil, 2006).

#### 2.3.2. Stances

One can frame explanations in terms of the stance that they take. Dan Dennett is known for drawing this distinction. Each stance speaks to a framing device for explanations. Each stance is general and non-predictive but does speak to certain relations, properties, and arguments that are fundamental to each (Keil, 2006). Dennett highlighted three different kinds of stances: (1) mechanical, (2) design, and (3) intentional. Mechanical stances consider only simple physical objects and their interactions. The design stance considers entities as having purposes and functions that occur beyond mechanical interactions. Some argue that teleology/functional explanation is part of this stance. There are also questions about whether an intentional designer is necessary for teleological explanations. The intentional stance sees entities as having beliefs, desires, and other mental contents/representations that govern their behaviors (Keil, 2006). These mental states then have causal consequences in terms of behavior. This has, however, often been criticized for being based on folk psychology (Woolman, 2013). Each stance describes different insights and distortions and explains different things. They need not exclude each other and can be complementary (see part G of the Supplementary material for more information on intentionality).

### 2.4. Causation

Causal explanations have been the most dominant explanation, especially in the sciences. However, these are not the only forms of explanations; there are also non-casual explanations, which are called constitutive explanations (Salmon, 1984).

#### 2.4.1. Causal capacities as explanada (etiological)

The object of constitutive explanation is the causal capacity of a system. This capacity describes what a system would do under specified circumstances/conditions (under a certain trigger). Causal capacities speak to what would, or could, or will happen under certain conditions and it includes notions such as ability, power, propensity, and tendency. Causal capacities speak to processes and events: when process (X) happens, event (Y) happens. These explain the changes in properties of a system—that is what an event is (Ylikoski, 2013). They focus on the origin, persistence, and changes in properties of (or in) a system.

#### 2.4.2. Counterfactuals and the Millian method of difference

This is the “Millian Method of Difference” (Encyclopedia Britannica, 2023) or counterfactual approach. Counterfactual explanations (Mertes et al., 2022) are the “knockout” kinds (the gene as the unit of inheritance was established through this approach). Here, if you want to determine whether something (C) as a cause has an effect (E), you perform an experiment whereby you remove (C) and then observe the effects. This can be a literal removal or a conceptual removal. This is often used to explain why something happened, such as a decision, event, or outcome by reference to a particular thing or sequence.

You can also change the values of (C) by making it stronger or weaker, and then observe what happens to (E). We use this to make inferences from the difference observed in effects where (C) is absent or different. Thus, we infer the causal role of (C) based on its presence versus its absence or its changes. This is effective for identifying discrete explanatory privileged causes (Walsh, 2015) (see Part A of Supplementary material for an example and information on its undesirability).

#### 2.4.3. Causation in complex systems

Complex adaptive systems can maintain stable configurations despite perturbations because they can alter the causal relations that happen between their parts. Each part affects, and is affected by, others, and the overall effect is attributable, jointly and severally, to all the parts. The system is thus affected by itself, and these causes are non-separable. Causes are only separable when the effect of a change in one is independent of the effects of changes in others. *If we remove or interfere with one we would also be interfering with others*. Therefore, causal composition/decomposition fails on non-separability—the influence/control factor of each part is non-determinable (thus non-quantifiable), and we cannot attribute differences in effect to specific differences in the causal contributions of the parts. One cannot assume when reviewing a result that the other factors are functioning as they were before the removal of a factor—they can be operating differently. Thus, we cannot decompose causes and differences in effect by reference to external versus internal influences. Changes in the dynamics of complex adaptive systems can be initiated endogenously through internal perturbations or exogenously through changes in the environment. The system mounts a response to both, and the result of that response is attributable to both internal and external influences as a single cause. Feedback is where the internal dynamics and environment both cause a change in the behavior of a system with signals. Thus, the environment *is part* of the system's dynamic structure. This is why it is difficult or impossible to attribute liability (either for an action or for a composition of a product or artwork) to either an AI system or a human, whereby there is a “commingling” between both. Even distinguishing between “principle causes” and “initiating causes” does not offer an adequate solution.

Complex adaptive systems tend to distinguish between “principal causes” and “initiating causes”. Principal causes are those to which we can attribute a large portion of the observable effect. Initiating causes starts the causal process, which ends with an effect. If two identical systems diverge in their outcomes, it is reasonable to afford principal causal responsibility for differences in effect to a factor that initiates the different trajectories (Walsh, 2015) (assuming that all other components contribute as before). In such a case, the principal causes would be initiating causes. However, the inference cannot hold for complex systems. There is logical discord between (1) the proposition that a change in the dynamics of complex systems is *initiated* by changes in exogenous conditions; and (2) the conclusion that *the principle cause* of the overall effect is *that change* in the exogenous condition. All this means is that the usual modes of inferences (cause and effect) do not work in complex dynamical systems.

### 2.5. Constitution

The constitution explains how things have the causal capacities that they do by relying on their parts and organizations (Ylikoski, 2013). Constitutive explanations ask: “what was it about (X) that resulted in it having disposition (Y)? What is it about (X) that enables a causation event to happen?” They provide different information compared to causative explanations. Fundamentally, these explanations provide modal information for causal possibilities.

To explicate constitutive explanations (Cummins, 1975, 1983, 2000; Craver, 2007a,b; Craver and Bechtel, 2007), their *explanada* must first be described. Constitutive explanations are not related to behavior, reactions, or activities of a system. These explain the properties of a system themselves. The *relata* of causative and constitutive explanations thus differ; causal explanations deal with events and constitutional explanations deal with properties. A constitutive explanation would say, for example, that system (S) has a causal capacity (C) in circumstances (E) because of its components (S1) and (S2) and their organization (O) (Ylikoski, 2013). Therefore, there is an ontological difference between causation and constitution. Both are relations of dependence (Rosen, 2010), but they are metaphysically different. Both, however, must account for explanatory relevance.

Metaphysics posits that the parts, their causal capacities, and their organization constitute the causal capacities of a system/whole. Constitution is synchronous and thus they are atemporal (meaning that it is not based on time and can be instantaneous). This means that if there are changes in the basis, there is an instant change in the causal capacities of a system (hence constitution is process and time-independent).

Importantly, the constitutive relata are not independent existences. In causation, one can insist that the relata of cause and effect are distinct from each other but one cannot insist on the same within constitution relata. Specific causal capacities are direct functions of certain constitutions. Constitutions then do not have independent identities.

Constitutive explanations distinguish themselves from identity, in that identity is a reflexive relation and is symmetric. First, one must distinguish between the constitution of all causal capacities of a system and the constitution of an individual capacity (Ylikoski, 2013). The former is the complete set of causal capacities of a particular system (at a time). We can identify the causal capacities and their causal basis (the organization). To have specific causal capacities, a specific causal basis (organization) is first necessary. Symmetries can be exact and help with allowing for simplicity in explanations, but it does not correlate to being the *identity* of a thing.

We cannot identify individual causal capacities with or as their composite bases (alternative constitution). This is because different objects can have the same causal capacities despite having different compositions (Ylikoski, 2013). This is known as multiple realization (MR). MR implies that we cannot equate a specific property of an object (like fragility) with the specific structural element of an object (molecular structure), but we can attribute a specific property of the object *because* of a specific structure that it has. At the heart of the scientific inquiry are questions about what makes causal powers possible and how changes in the organization of parts affect the total causative capacities of the system. Science largely involves studies of constitution (the study of the relation of dependences). Therefore, the constitution is at the heart of causal inquiries. There is justification then for an approach of the constitution to explain agent status or agency. This explanation offers a method for granting an AI system agent or person status through relations of internal dynamics and dependencies. This understanding of the constitution is what Kant was alluding to in his oft-quoted notion that things are to be understood as ends in themselves and never as means to an end.

The necessary asymmetries are present; the constitution explains causation, and the constitution is composed of parts and the organization of those parts. Systems then are made of causative parts and their organizations. The other asymmetry is existence. This asymmetry means that parts can exist independently of systems, while systems cannot exist without their parts (they can exist without some parts, but not all). The organization of parts is also fundamental for maintaining the status of a system (since systems are not reducible to their parts, they are greater than the sum of their parts). Organization therefore has explanatory relevance. Systems' causal capacities are not just the sum of their parts; they are also the organization of those parts. Organizations' explanatory relevance stems from their contribution to the causal capacities of the system as a whole (change organizations and the causal capacities of the systems change). Organization is also called contextual causation and is empirically observable. Contextual is similar to downward causation (below), except that it displaces the notion of “downward” and instead posits that parts can influence each other regardless of a relative placement in relation to each other (Ylikoski, 2013). Parts can be of different sizes, different levels of abstraction, and situated at different levels. Causation is not limited to agency nor human agency, but it can also include instances of manipulation/intervention.

Constitution and causation are both explained in terms of their dependencies, which are a particular set of “objective” relations of dependent facts. These facts give explanations a direction and they are the basis for explanatory preferences (explanations must explain the systems' causal capacities in terms of their basis and not vice versa) (Ylikoski, 2013). Constitutive relations involve causal manipulation.

### 2.6. Downward causation

Downward causation provides an explanation for “emergence” which will also be necessary for an explanation of AI agency. However, downward causation has been criticized. For example, Kim (2006) argues:

“[d]ownward causation is the raison d'etre of emergence, but it may well turn out to be what in the end undermines it”.

However, this argument assumes the causal inheritance principle, which stipulates that the causal powers of complex systems are inherited exclusively from the causal powers of their parts. This has two salient points: (a) If parts do not have causal capacities, then the system as a whole would not (the capacities of the whole counterfactually depend on the capacities of the parts); and (b) in complex entities, nothing other than their parts are relevant to the determination of their causal properties. This then requires the causal powers of an entity to be internal to it.

Internal properties are context-insensitive, and an entity/system has all its internal properties (until there is an internal change) regardless of the context. If causal powers are internal, it is only the internal constitution of a system that confers those causal powers. This, and the assumption of internal causal properties, results in an ontological primacy afforded to the capacities of the parts, as opposed to the capacities of the totality/aggregates. The idea is that complex entities inherit their causal powers from their parts, but that the converse is not true. Complex entities cannot confer on their parts' causal powers which the parts did not have by their internal natures/capacities. Therefore, the properties of complex entities cannot explain why their parts have their causal powers (Walsh, 2015). This is Kim's argument against reflexive downward causation.

Kim's argument against emergence is the assumption of internal causal properties. This kind of thinking may have arisen from the notions of how mass (as a fundamental causal power/property) is context-insensitive. Masses of macroscopic objects are not altered by the masses of other bodies; mass behaves in a context-insensitive manner with regard to forces. An object's mass allows the prediction of its behavior across different contexts where forces act on it. It allows for the assumption that their effects are mutually independent, but do not affect masses.

Context insensitivity of causal powers is present in the analytic method (Cartwright, 2007). The assumption is that as contexts are altered, entities' causal powers remain unchanged because of the internal nature of the causal powers. However, context insensitivity does not equate to internality. The mass itself shows this. It is possible for a mass to be invariant across many contexts, but it is not an internal property of a body. For example, recently, it was discovered that the mass of protons comes from a combination of the masses of their constitutive three quarks, their movements, the strong force that ties them together (the gluon), and the interactions of quarks and gluons (Thomas Jefferson National Accelerator Facility, 2023). Hence, the mass of the proton is emergent. Mass is conferred onto a proton by its relations to something else. Causal powers may therefore be invariant in different contexts; they can be relational properties of things.

If causal powers are non-internal properties conferred on things by contexts, then one can argue that parts of complex systems get their causal powers from the system as a whole (*connubiality*). The parts would not have those capacities if they were not parts of that complex entity. The whole system, in this way, is the context that confers causal powers on its parts. This holds true even if the causal powers of a whole system are completely inherited from its parts.

Therefore, the property of the whole depends on the properties of the parts, and the converse is also true. If properties are understood to be relational (not internal in the strictest sense) and context-sensitive, it becomes easier to understand. Reflexive downward causation can be explained as follows: If they are relational properties, it means that complex systems have the causal powers that they do because of the causal powers of their parts (as in causal inheritance). It is also possible that parts have their causal powers because of the complex system they are part of.

In causally cyclical systems, one can assume that the causal powers of the parts are context-dependent and are conferred by the system in which they are parts. Hence, *emergence* is a fact of complex systems which can transform their parts (Ganeri, 2011). By transform, I mean that they confer on their parts capacities that they counterfactually would not have. These capacities reciprocally fix the properties of the system. Therefore, emergence can arise based on the context. Systems can give their parts causal powers and causal powers of the parts can be explained through reference to the system as a whole and its properties. They are hence relational, and the more suitable framing of this would be “intrinsic” as opposed to “internal”. This is developed further at the end of the article.

### 2.7. Fundamental and emergence

“Fundamental” speaks to things that cannot be decomposed further into smaller resolutions, meaning that we cannot get a coherent theory if we do so. What is fundamental is thus contingent on knowledge and the era that you find yourself in. Previously, atoms were thought to be fundamental until particle theory was established. However, emergence is different from fundamental, and unlike fundamental, emergence is something that is not conceptually contingent in the same way that fundamental is. “Emergence” can explain many issues in physics, such as how Schrödinger's (1944) order-from-disorder answer in his book *What is Life?* gives us a hint of a theory that incorporates emergence into complex systems. Out-of-equilibrium systems, for example, spontaneously build structures that dissipate energy, and, as they do this, they become increasingly stable and more complex. They have their own intrinsic dynamics. The dynamics of these systems can yield predictions and explanations, not just about the activities of the whole system but also about the activities of the parts. The movement of information toward order is also an emergent property. Vopson and Lepadatu (2022) demonstrated that while the thermodynamic entropy of systems increases (in terms of the studied virus), the *overall* informational entropy decreases (or stays constant). This is “the second law of infodynamics”. The law itself works in opposition to thermodynamic entropy; it describes this movement as an *emergent entropic force*. Thus, we can now account for (1) informational emergence in complex systems, which (2) are not considered to be “alive”.

### 2.8. Variance

#### 2.8.1. Multiple possible variance and rarity

The microstate of a system is the configuration of the system (Hidalgo, 2015). Entropy is the logarithm of the fraction of all equivalent states. Entropy is lowest where the states have the least possible variance (order), and it is at its highest where there is the most possible variance (MPV). Rarity (Hidalgo, 2015) is the measure of the possibility of a particular arrangement occurring at random or without intervention. If it is rare, the probability of it occurring *without intervention* is unlikely. Functionality and working conditions are indicators of rarity. The natural state of things is to be in disorder, as opposed to order. States of the disorder have less information, and thus, the destruction of a physical order is also the “destruction” of information (informational content). Creating physical order is creating information (which is embedded in that order). The rareness of a state of order is measured against the number of possible states. One manner to do this is by correlating the connections between states. There is a correlation if one can get from state A to state B with a simple transformation. Information-rich states that involve correlations give the word “information” its colloquial meaning. Most things are made up of information. “Order” is a statistical probability measure of occurrence. Sometimes, the states of systems do not allow changes from A to B, or they impose limitations on the modes of transformation. The modes of achieving disorder outnumber the modes of achieving order.

#### 2.8.2. Covariance, correlation, and mutual information

In statistics, correlation describes *the degree* of *linear dependence, association, distance, or relation* between two random variables in data. Correlations and standard deviations apply only in mediocristan non-scalable environments (Taleb, 2007) (Gaussian or bell-curves) wherein magnitude does not matter. In other words, both only have predictive or informational power in that context (see part D of Supplementary material). They can only be used to draw *qualitative inferences*.

Importantly, while correlation applies only to *linear relationships* between variables, this linear relationship (the signal) between variables *does not scale linearly*. Correlation is not additive (Taleb et al., 2023) because the correlation coefficients are non-linear functions of the magnitude of the relations between variables. They cannot be averaged for this reason. In turn, this means that an average of the correlation coefficients does not equal an average correlation itself. For example, a correlation coefficient signal of 0.7 conveys much less information than a coefficient of 0.9, while a signal of 0.3 conveys almost the same relationship as that of 0.5 (Salazar, 2022). Correlation cannot be used in non-linear relationships between variables, which is what characterizes reality (or Extremistan, scalable environments). Using it here will result in an incorrect explanation of the relation between random variables. In short, correlation does not accurately reflect the *informational distance* between random variables (Taleb et al., 2023).

Covariance speaks to the linear measure of the strength of the correlation between two or more sets of random variables. The covariance for two random variables (X) and (Y) each with a sample size of (S) is defined by an expectation value (Weisstein, 2023). Where there is a correlation between the values, the covariance will be non-zero. Where they are not correlated, it will be zero. The covariance can be directly proportional or inversely proportional. Covariance can be infinite, while correlation is always finite (Taleb, 2020). Covariance provides a method for construing features of contexts as “affordances” since this would be a qualitative finding and one that is non-scalable as described below.

The appropriate measurement function is *mutual information* (MI), which is not dissimilar to the Kelly criterion in finance and risk (see Supplementary material). In machine learning, this is known as *relative entropy* and is based on the expectation of the Kullback–Leibler divergence (a measure of similarity between distributions) (Taleb et al., 2023). Machine learning loss functions rely on entropy methods. Mutual information can be understood as a non-linear function of correlation; if mutual information increases, correlation itself increases, *but non-linearly*. Mutual information compares the probability of observing two random variables together with the probability of observing those same two variables independently (Prior and Geffet, 2003). In other words, an MI approach captures non-linear relationships and, importantly, it also *scales to noise*. The MI approach describes the amount of mutual dependence between two random variables; one gains information about a random variable by observing the value of another random variable. It measures this amount of dependence in information (in bits) and is used in instances of non-linear dependencies and discrete random variables. This is an entropy measure, *and it is additive* (Taleb et al., 2023). This understanding of how “seemingly” random variables are related in terms of how the values or changes in one variable affect the understanding of the values or changes in another is an important tool.

Mutual information maps to the mutual dependence of random variables (how much can I rely on X if I know Y). Therefore, an MI approach would be most applicable to genetic distances (Taleb et al., 2023). Furthermore, an information metric is preferable and suitable for an account of agency or personhood, since DNA is understood as the basis of “life”. Mutual information then provides the proper tool for creating a methodology with proper scaling, proper explanatory value, minimal informational loss (Taleb et al., 2023), and avoidance of using linear approaches (such as Cartesian methods or internal–external measures). Conditional mutual information (also known as transfer entropy) provides a suitable manner for causality detection since non-linear relationships (Mukherjee et al., 2019) in data associated with genetics and biological systems make *generalized data impossible*. Transfer entropy provides a consistent method across different conditions.

#### 2.8.3. Intervention

Interventions usually involve notions of manipulations carried out on a variable (X) to determine whether changes in (X) are causally related to a variable (Y). However, any process qualifies as an intervention if it has the right causal characteristics, and not just human activities (Woodward, 2000). Consider this example: First, there is an intervention (I) on variable (X) which is a causal process that changes (X) in an exogenous way. If a change in (Y) happens after this, this change occurs *only because* of the change in (X) and not because of another set of causal factors (Woodward, 2000). One must also define what intervention means; interventions involve exogenous changes that break or disrupt previously existing endogenous causal relationships between variables and system states. This understanding of intervention allows for an extrinsic manner of specifying intrinsic features. It allows us to distinguish between types of correlations and dependencies that reflect causal and explanatory relations and those that do not. Viewing intervention in this way also transparently allows for the epistemological designation of experimentation as the establisher of causal and explanatory relationships. This allows us to make claims about the role behavior plays in causality through the use of interventions (Woodward, 2000). This is a much clearer account of causation and explanation as opposed to the traditional doxa.

#### 2.8.4. Invariance, generalizations, and laws

According to Woodward, generalizations can be used in explanations and depend on invariance rather than lawfulness (Woodward, 2000). A generalization describing a relationship between two or more variables is invariant if it is stable or robust after the occurrence of an intervention or change in various other conditions at an appropriate level of approximation (Woodward, 2000; Maher, 2006). Invariance comes in degrees, and it has other features that capture the characteristics of explanatory generalizations in the social sciences, in particular (Woodward, 2000). In other words, invariance does not appeal to laws for its usefulness in explanations. The set or range of changes over which a relationship of generalization is invariant is known as its *domain of invariance*.

There are two types of changes, and both are fundamental to explanatory powers. The first is changes in background conditions (changes that affect other variables other than those variables which are part of the generalization) (Woodward, 2000). The second is changes in variables that are present solely within the generalization itself [within the Newtonian equation of *F*=*ma*, the change can occur to mass as (m) or acceleration as (a)].

For a methodology to constitute a law on personhood or agency, it must meet the conditions of laws (see part A of Supplementary material). This includes being a generalization with a higher invariance or wide applicability and being confirmable, predictable, and integrable (not only including being integratable with other laws, but also with philosophical or jurisprudential axioms which may ground legal laws, such as Kantian philosophy). Laws can also replace other older laws where they demonstrate that the older laws were unsuitable or provide less information.

#### 2.8.5. Explanations and invariance

Good explanations require the use of invariant generalizations, which enable the specification of systemic patterns (of counterfactual dependence). This converts information into explanations since it can be used to answer a range of counterfactual circumstances about the explanandum. This allows for better predictive models. There are various kinds of counterfactual dependences, including active and passive ones; active is the type that is necessary for good explanations (Woodward, 2000). Invariance is thus necessary for reliance on counterfactuals and prediction (and to some degree also causal links). Invariance comes in degrees. There is also a connection between the range of invariance and explanatory depths; generalizations with more invariances constitute better explanations, especially for science. Generalizations that are not invariant under any conditions have no explanatory powers. Invariance is also important for building a purposive teleological account and countering the notion of “chance”.

### 2.9. Theories of explanation: teleology and mechanism

#### 2.9.1. What is teleology?

Teleology explains the existence of a feature based on its purpose (Walsh, 2015). The understanding that biological organisms are self-building, self-organizing, or adaptive suggests that they are greater than the sum of their parts. Thus, we can argue that organisms are purposive things. Refer to Sommerhoff (1950) in part B of the Supplementary material for information on how capacities can serve as a criterion of purposiveness.

#### 2.9.2. Mechanism vs. teleology

Mechanists argue that natural selection explains the fit and diversity of organic forms, thus making teleology or purpose explanations unnecessary. The mechanical view is that every event has a cause, with causes being able to fully explain events. But there are three main arguments against this approach: (1) non-actuality, (2) intentionality, and (3) normativity (Walsh, 2015).

The non-actuality argument states that means come before ends (goals). However, in terms of teleology, ends *explain* their means. Therefore, teleology in this light is inferential: it is the process of positing one's own presuppositions to establish an end. When the means occur, the goal or ends are not yet realized (they are non-actual). How can a non-actual state affect or cause a means?

The intentionality argument states that non-actual states of affairs cannot cause anything but mental representations of them can. One way to solve the teleology non-actual dilemma is to propose mental states as representations of these goals (or ends). Thus, occurrences of actions or events are explained by intentions as mental states of agents. The intentional and mental state argument is the most common justification of teleology (Kant and Bernard, 1790). The issue is that organisms typically do not have intentional states. However, this intentional and mental state justification is most commonly used in teleology. The earliest form of teleology can be found in Plato's *Timaeaus* and in the works of Thomas Aquinas; after all, any perceived forms of an order must presuppose a purpose or an intention. Aquinas argues that whatever lacks intelligence cannot move toward the end unless it is directed by knowledge, “as the arrow is shot to its mark by the archer.” Intentionality is the obvious paradigm for teleological framing. Kant (2000) notes that intentionality is our only model for understanding purpose.

The normativity argument suggests that teleology has a normative value. Explaining an action as a consequence of intention is to argue that an agent was rationally required or permitted to act in a particular way to achieve certain goals. Rational actions are those which are required to attain a goal (or end). Thus, a teleological approach must account for an action being rational (Walsh, 2015).

Bedau (1991, 1998) argues that because of the normativity of teleological explanations, goals can have their explanatory roles only if they have intrinsic normative properties. Namely, (*c)* construed as a means toward attaining a goal *(g)* could only be something that a system *ought* to produce, if (*e)* is a state that the system *ought* to attain, but (*e)* could not be an “ought to attain” state unless (*e)* was intrinsically good. The issue is that natural facts are not intrinsically evaluable (Walsh, 2015). A proper account of teleology must account for all these arguments in making space for purpose. Furthermore, a proper teleological account must not be purely metaphysical, but must also operate within a scientific framework. Emergence is an important aspect of the account of agency. The dynamics of agents must be explained by their purposes and affordances. These would be emergent properties that emerge from the relation between agents and their contexts. They are not properties of the systems' parts themselves. Mechanistic explanations tend to exclude emergence since they appeal to the dynamics of complex systems as being entirely explainable through the properties of their parts (Walsh, 2015). Parts are not emergent. However, before solving the emergence issue, I need to account for “purpose”.

#### 2.9.3. Teleology and purpose

Teleology explains the existence of a feature based on its purpose (Walsh, 2015; Kampourakis, 2020). We can argue that organisms are purposive things because organisms or agents are self-building, self-organizing, and adaptive, which suggests that they are more than the sum of their parts.

#### 2.9.4. Chance and purpose

In biology, Jacques Monod considered the consequences of a non-purposive nature/biology. He identified a contradiction at the heart of evolutionary biology. This is the “paradox of invariance” (Monod, 1971). The paradox is that living creatures show two contradictory properties: invariance and purpose. Invariance is the ability to reproduce and transmit information, including *ne variateur* information. *Ne variateur* information relates to its own structures and is transmitted from one generation to the next. The purposiveness of organisms is evident in the maintenance of their viability by responding to environments and adaptation. However, many would argue that science does not recognize this kind of purpose because it seems to be a contingent truth instead of an objective one. To explain this, Monod suggested that purposiveness can be explained by the mechanism of molecular invariance (Walsh, 2015).

However, the invariance principle raises complications as evolution is fundamentally about change. Adaptive evolution is a form of environmentally charged biased change. Thus, there should be a source of new variants and a process that is biased toward change. If we argue that new variants are biased in favor of goals and purposes, we may also be undermining science. For Monod (1971), the source of evolutionary novelties must come from unbiased chance. Monod argues that chance must have a requisite role in evolution, and this role is methodological and not metaphysical. This is akin to Democritus, who argues that everything is a result of chance and necessity. With chance and necessity, there is no need for purpose (Walsh, 2015). However, the chance is unsuitable for an account of purposiveness that I want to build.

Aristotle took issue with Democritus's explanation, since chance is, by its nature, not measurable. In *Physics Book II*, Aristotle discussed what an explanation should include. His arguments were developed to counter the atomists' arguments at the time, which are similar to the mechanists' arguments of cause and effect. He did not like explanations that did not account for something—and chance was unaccounted for. He illustrates this (*Physics II.5*) (Barnes, 1991) with the story of a man who is collecting money. The man meets a debtor at the market and collects money owed to him. This, for Aristotle, is a chance encounter since the collector went to the market for a different purpose; he coincidentally also collected his money. This is a mechanistic explanation, and these explanations do not distinguish between occurrences that are regular/purposive or chance. They therefore give incomplete information since they do not distinguish between both. Mechanistic explanations are necessary since every occurrence must have a mechanical cause, regardless of whether it occurred for a purpose or because of chance (Walsh, 2015).

Purposive events are, however, robust (invariant) across a range of alternate initial conditions and mechanisms, whereas chance events are not (they have differing modal profiles). Good explanations must be able to distinguish these. Purposive encounters are those which are insensitive to initial conditions, including locations. Thus, in purposive occurrences, the means counterfactually depend on the ends. Chance occurrences are sensitive to initial conditions and, if the initial conditions are different, the event or ends would not have happened. Unlike chance occurrences, purposive occurrences are sensitive to goals. If an agent's goals were different, the event would now have occurred. If the collector had been elsewhere in the market, then the encounter may have happened elsewhere, at a different time, and by different mechanisms.

Given the counterfactual dependence of mechanisms and ends, events that happen because they serve a purpose can be explained in two ways: (1) the occurrence results from mechanical interactions and (2) the occurrence is conducive to the fulfillment of a goal. However, one thing is certain; one cannot simply disregard purposes. If purposes are ignored, it induces a “selective blindness” to a class of explainable occurrences, namely, those that are structured according to the counterfactual dependence of means on goals. This is not just an error of omission; it also risks misconstruing purposive occurrences as blind chance. To properly account for events, both teleology and mechanistic explanations are needed. I have now explained purposiveness as goals; these purposes can also explain their own means (Walsh, 2015).

#### 2.9.5. Goals

Goal-directed processes are those that are conducive to stable end states and their maintenance. The end state itself is the goal. Thus, a goal is a state that the goal-directed process is aimed toward. Central to studies on natural goal-directed processes is an adaptive and autonomous system, which can achieve and maintain persistent and robust states through the implementation of compensatory changes (Di Paolo, 2005; Barandiaran et al., 2009). These systems can pursue goal states and sustain them in the presence of perturbations. They can effectively implement changes to component processes in ways that correct the effects of perturbations, which could otherwise result in the system not achieving its goal (Walsh, 2015). This will be necessary for an account of purpose and agency.

The architecture of the system underpins the goal-directed capacities and states of the goal itself. These systems are usually comprised of modules. These modules are clusters of causally integrated processes decoupled from other modules. They also demonstrate the capacity to produce and maintain integrated activities across a range of perturbations of influences (robustness). Each model has regulatory influence, using positive and negative feedback, over a small number of other modules. Each part effectively influences other parts in some way. This allows for robustness and plasticity by maintaining stability in the presence of perturbations by enacting new adaptive changes. Robustness describes a property of something which can produce novelty, in response to novel circumstances. Biological organisms display this. What allows organisms or systems to do this is the modularity of their development (Schlosser, 2002).

Thus, goal-directed behavior is a causal consequence of the architecture of adaptive systems. Furthermore, it is an observable feature of systems dynamics. It is the capacity of systems as a whole to utilize the causal capacities of their parts, and the ability to direct them toward attaining a robust and stable end state. That end state or goal is not a mysterious something; it is a complex and relational property—the property of being in a state that a goal-directed process can achieve and maintain. Therefore, goals are natural and observable (Walsh, 2015). Goals are thus not “mental states” and instead are naturally derived from a system's intrinsic dynamics.

But what about the content of teleological explanations? We can determine the conditions under which they apply as explanations, but we must also account for the content of the explanation. There is a fundamental difference. Conditions for teleology can be understood as causal occurrences; however, content cannot be described in causal terms. Teleology is not about explaining causes, it is about explaining goals to which events are conducive (Walsh, 2015). Thus, for agency, we no longer need to rule out an entity based on being “created” or “developed” by something or someone else. The focus is on the entity itself.

#### 2.9.6. Teleological explanations and invariance

To describe a non-mechanistic account of goals, two questions must be answered: (1) How can an event be explained by citing the ends to which it is simply a means; and (2) Why does this explanation not need to be explained through mechanisms of cause and effect?

To address the first question, goals can explain their means of achieving those goals in a way that is similar to how mechanisms explain their effects by using counterfactual invariance relations. Invariance here does not mean the transmutation of stability of form across generations or lineages. Here, it is *Woodwardian invariance*. We can answer the second question by simply demonstrating that they appeal to different invariance relations more than mechanistic explanations do (Walsh, 2015).

Mechanistic explanations demonstrate how activities and characteristics of (X) produce (Y) as the effect including the specific properties related to that effect. Activities *produce* effects, which are related through the notion of counterfactual dependence—effects counterfactually depend on their mechanisms. These activities can be expressed in terms such as “binding”, “opening”, and “bending”. Woodward (2003) called this “relation invariance”:

“[T]he sorts of counterfactuals that matter for purposes of causation and explanation are just such counterfactuals that describe how the value of one variable would change under interventions that change the value of another. Thus, as a rough approximation, a necessary and sufficient condition for *X* to cause *Y* or to figure in a causal explanation of *Y* is that the value of *X* would change under some intervention on *X* in some background circumstances”.

Thus, we can use this to explain how events as means are related to their goals. If there is goal (X), which then produces event (A) which is conducive to (X) under conditions (Q), then under different conditions (V), it would produce event (B), as (B) would be more conducive toward (A). If the system had another goal (Z), it would produce event (C), should (C) be more conducive toward attaining (Z). This is an invariance relation. It is the obverse of the relation of cause and effect. In other words, we explain that causes themselves explain their effects, because when the cause occurs, then so too would the effect. If the cause does not occur, neither does the effect. We can also reason that a goal explains its means because if a system has a goal then the means too would arise, and if there was no goal then the means would not arise.

This explains how events, as means, are related to their goals. Causes explain their effects because when the cause occurs, so does the effect. If it does not, neither does the effect. We can also reason that a goal explains its means. If a system has a goal, the means arise; without a goal, the means do not arise. But, on its own, invariance is insufficient. Explanations are description-dependent, and good explanations enhance understanding. Mechanistic explanations do not simply speak to cause and effect (relations), and they also speak to the appropriateness or accuracy of that relation. The relation itself only exists if it is appropriate. We use concept descriptions such as “push”, “pull”, and “attract” to describe productive relations. These speak to the nature of the relation, and sometimes also explain the effect.

For teleology, we use the concept descriptor of “conduce/ive”. So, the modal relations are (1) causes produce effects; and (2) means are conducive to their ends. *Conducing is not causation*. A means is only considered conducive to its ends if it robustly and reliably brings about the end *ceteris paribus* across a range of counterfactual circumstances. Hence, if the goal is (A) and event (X) causes (A), this does not mean that (X) conduces to (A) (Davidson, 1980). Thus, producing and conducing are descriptions of events, and they have different informational content. Producing specifies an earlier event (time is important here), which is the mechanism for the later event. This describes *how* the later event arose. Conducing specifies the *why* of an event—that it is conducive to realizing or maintaining a goal.

A singular event can be explained in terms of mechanistic (causal) and teleological (conducive) relations. The former explains how things happen, while the latter explains why they happen, and thus they co-exist. They are complementary and non-competing. They are also complete—they do not need each other to explain their own coherence—the how's explain the how's and the why's explain the why's, and we do not need the how's to explain the why's. They both explain different information about events. However, for the completeness or coherence of an explanation as a whole, one needs both types of sub-explanations. Without both, there is an explanatory loss. Thus, both mechanism and purpose are important for explanations but not for independent systems themselves. The non-actual claim, for example, is a conflation between causes and explanations. In terms of the intentionality counter, intentions can be understood as goal-directed activity instead of mental representations. Intentional states are mental representations and are unnecessary for teleology (Walsh, 2015).

In terms of the normativity counter, the goal need not be described as “good” to explain why systems *ought* to act in certain ways, which result in conducing to that goal. Systems will do what it takes to achieve the goal; there is no specific modality to be followed. The modality need not be prescribed, singular, or of a specific nature (such as good or valuable). What matters is *appropriateness*. There is thus no need for an evaluative state of affairs. Aristotelian teleology is not intentional, transcendent, or causation-based. It comes about because of the activities of goal-directed entities which are observable and occur in the natural world. This can be used for both predictive power and explanatory power in the same way that we use other robust regularities (Walsh, 2015).

### 2.10. Theories of explanation: agents and objects

#### 2.10.1. Natural agents

Natural agents are obtained from the natural purpose explanation. Agency, such as purposiveness, is an observable property of a system's gross behavior. The system can pursue goals and respond to conditions of its environment and its internal constitution in ways that promote the attainment and maintenance of its goal states. The agency is observable in the sense that we see agents negotiating with situations using its dynamics. We can see a range of robust and regular responses to conditions. If we understand its goal, we can understand its behavior. The agency is ecological as a system that can cope with its context and achieve its goals by responding to *affordances as affordances*. An ecological definition of the agency includes three inter-definable factors: (1) goals, (2) affordances, and (3) repertoire (Walsh, 2015). Affordances are opportunities for, or impediments to, a goal; only goal-directed systems can experience its conditions as affordances. Systems can experience affordances only if they have repertoires, which are sets of possible responses that systems can enlist in pursuit of goals (in response to the system's experienced conditions). For repertoires to constitute a response to affordances, repertoires must be biased. Systems must be able to exploit behavioral repertoires in response to conditions in ways that are conducive to the attainment or the maintenance of their goal. The goal of the system is the state that it moves toward attaining/maintaining by directing behavioral repertoires in response to affordances conducing that state. Repertoires come in degrees, and some agents have richer repertoires than others. Systems with wide ranges of repertoires can respond to more affordances and can pursue a wider range of goals. Ecological agency is not all-or-nothing: It comes in degrees. There is a continuum from the most basic agents capable of pursuing a narrow range of goals to those possessing greater repertoires of responses. Cognitive systems tend to have large repertoires, with thinking forming part of their repertoire (Walsh, 2015). This is a model in which we can “grade” or rank the agent status of a system. A system will have a greater agent status grading if it demonstrates a greater repertoire (as variable responses to affordances) for maintaining or improving the conduciveness toward a goal (see Parts E and F of the Supplementary material).

#### 2.10.2. Object and agent theories

There is a difference between object and agent theories. Object theories that we use today aim to describe and explain the dynamics of objects (Walsh, 2015). To construct these theories, we create a space of possible alternatives for those objects. This is known as a “state space”. We then look for principles that may account for various possible trajectories through this state space. The objects in these domains are subject to forces, laws, and initial conditions. Lee Smolin dubs this the “Newtonian paradigm” (Smolin, 2013). This describes system dynamics by the answers to two questions: (1) What potential configurations does the system have; and (2) In each configuration, what forces is the system subject to (Smolin, 2013)? In this paradigm, the laws, forces, and initial conditions are irrelevant to and exist separately from the objects. Object theories are transcendental, and they have an explanatory asymmetry. Transcendental means that the principles that govern the dynamics of the objects in the theory's domain are not part of the domain itself. They do not evolve as the system does, and the laws of nature and the space of possibilities through which the objects move remain constant as the objects change (Walsh, 2015). This allows for the explanation of the changing state of a studied system by appealing to unchanging laws.

#### 2.10.3. Action theories

The Cartesian view holds that agents' thoughts, beliefs, and desires explain their actions only if they cause said actions (Davidson, 1963). This means that contemporary action theory is interpreted as implying that thoughts are mental entities realized as internal physiological mechanisms and that these mechanisms combine with other internal mechanisms to effect actions. They do so by their intrinsic causal properties (Fodor, 1987). Actions are outputs, such as an internal process of computation, and they result from the mechanical interactions of the internal states of the agent. The purposes of agents and their dynamics do not appear in the explanations of actions. The Cartesian model (thought and action) posits that agents are akin to “middlemen” (Walsh, 2015) since they are the connection between the causal activities of their psychological states and the environmental demands that they experience (Walsh, 2015). Haugeland (1998) described the notions of *intimacy* and *commingling*. His conception was in opposition to the Cartesian mind which posited that the mind is entirely internal to the agent, and the position that the environment is entirely external to the mind. In Cartesian dualism, both communicate through perception (which is environment to the mind) and action (which is mind to environment). Haugeland (1998) thus argued that the mind played an active role in constituting the environmental conditions to which it responds. Intimacy in this explanation described the mind as embodied and embedded in the world. This is not just an interdependence but also a “commingling” or integralness of the mind, body, and world, which undermines any separation between them.

#### 2.10.4. The disappearing agent

The standard action theory approach created the issue of the missing agent, which is a consequence of its underlying methodological commitments (Velleman, 1992; Hornsby, 1997). These have arisen from precepts of the Cartesian mechanisms already described. It ignores the fact that actions do not happen to agents: *they are performed by them*. Cartesian mechanisms of action miss this point—an action is something produced by an agent for a reason. A proper account of action involves explaining the doing of agents by highlighting them to be reasonable or rationally justified considering the agent's purposes. The agent's goals will explain the appropriateness/conduciveness of the actions undertaken. Viewing actions as just causal consequences of internal states erroneously misses the fact that actions are purposive activities in lieu of goals. The Cartesian object theory views agents as objects, wherein the actions of agents are explained/caused by extraneous forces that act on said agents. It does not explain actions as products of agency, but rather as effects of extrinsic causes: external environments and internal computation and representation. Thus, it is an exclusion of agency which is both real and natural. This is also present in the understanding of “rational action”. Action theory is divided between two conceptions of humans: (1) as objects in the natural world, subject to external causal influences; and (2) as agents able to initiate actions that are guided by reasons (Walsh, 2015).

Merleau-Ponty explains behavior as commencing with an active organismal agent that is problem-solving and goal-pursuing (Matthews, 2002). The agent responds to conditions as meaningful, either obstacles or opportunities. The goals and capacities of the agent give importance to the conditions. Thus, actions are responses initiated by agents to sets of affordances, and these affordances are largely of the agent's making. Agents also co-evolve with these affordances in line with their actions and goals. Agent theories of actions view actions as events that are generated by agents because of agents' pursuit of the goals. These purposes explain and justify the actions and not the other way around. Adaptive evolution is thus a phenomenon of agency. Thus, using an agent theory of this sort enables proper conceptual underpinning for agent status and agency in combination with natural purpose and goals (see Part F of the Supplementary material).

#### 2.10.5. Autonomy

Agents create degrees of freedom for themselves by constituting their affordances through self-maintaining and self-regulating activities. They determine which environmental conditions are important. They also enable the exploitation of opportunities that the environment presents. This is a stronger account of autonomy. The integral processes in autonomous systems are (1) continually dependent on one another in their formation and realization as a network; (2) make up a unity (converge) in their domain of existence; and (3) govern areas of exchanges with the environment (Thompson, 2007). Autonomous agents can “make sense” of circumstances. Making sense means to detect and use the features of one's context, which in turn also constitutes the features/context. This is then the capacity of the agent to mobilize its resources in a way that supports the pursuit of its goals, and by exploiting opportunities or reducing impediments. Agents make features significant in the way they are detected and responded to in pursuit of their goals. In this way, autonomous agents construct and constitute the conditions that they respond to. There is a reciprocity of form and affordance—as form evolves so do affordances (Walsh, 2015). As mentioned above, this is related to the repertoire of capabilities. Thus, systems as agents that demonstrate a greater ability to identify, interpret, utilize, and implement features as affordances in pursuit of their goals would be graded higher [see Part C of Supplementary material for a supportive moral perspective on AI and agency and the supportive novel Technological Approach to Mind Everywhere (TAME) framing].

## 3. Constructing the AI agent

### 3.1. Write-re-write systems: semantic closure

Semantic closure is a concept that refers to the fact that a system can enclose meaning within itself. In biology, for example, a string of DNA and messenger RNA (mRNA), the encoding mechanism between both, has evolved, altering the meaning of DNA by rewriting the genetic code (Clark et al., 2017). In biology, the most important factors related to this concept are the ribosome, transfer RNA (tRNA), DNA, and mRNA (Clark et al., 2017). The tRNA is involved in expression which defines the meaning of DNA by mapping the three bases of DNA to one amino acid. Changing the mapping also means rewriting the genetic code. Hence, the meaning of the genome can itself be altered (Clark et al., 2017). Rewriting in biology is the process of moving from one semantically closed state to another.

It is important then to understand how meaning originated for translating proteins and how it has been altered through evolution. This is an ontogenetic or bottom-up approach (Clark et al., 2017). For this process of moving from one semantically closed state to another, there must be a necessary structure. Von Neumann was the first to describe what an artificial architecture that enables semantic closure would look like. His constructor theory birthed the modern form of universal constructor architecture (Clark et al., 2017). Some of these models have highlighted the necessity of redundancy in maintaining stability in the presence of mutations. In the proposed theorem of chemical construction theory, the authors also highlight the self-referential nature of the genome (it contains descriptions of all other machines in the system, and hence it is its own description) (Clark et al., 2017). In their experiments, the authors demonstrated how alterations in the expressors can lead to novel interpretations of the genome which, in turn, gives rise to pleiotropic effects. Thus, the meaning of the genome has been changed, and this new interpretation of it extends to other molecules, not just the expresser. They also demonstrated that it is not genetic material that evolves but also the mechanisms of copying. Each string can play different functions in many different relations or reactions. Control in this way is distributed throughout the system (there is no explicit or centralized control mechanism). The authors also postulated that the ribosomes may be the biological equivalent of any string that imposes meaning into the system (Clark et al., 2017).

Finally, the authors proposed something interesting: there were *emergent* or transient changes that were expressed and *which did not* appear in genetic records. *These arise through inaccurate expressions*. Their results demonstrate that these “errors”, while not reflected in the genome, are reflected in heritable changes in expression (they are covert). Errors in expression in biology are deleterious or non-heritable, since only genomic information is thought to be heritable (Clark et al., 2017). They also provide evidence for misreading errors of this nature, including the streptomycin-dependent phenotypes of *E. coli*. Errors in ribosomic interpretation of DNA have been demonstrated previously (Clark et al., 2017). In this way, they can change meaning. The authors stated that expressors can make a consistent interpretation of a genome (meaning it leads to its own expression). By interpreting its own genetic material, *expressors obtain meaning through self-reference*. From this, we can use semantic information as the central measure for an account of personhood or agency. Importantly, it is not tied to a biological brain, and systems can themselves enclose and change their own semantics. Self-reference in this light provides another framing for personhood and agency. This study also provides backing for “emergence”.

### 3.2. A semantics model for personhood, agent, and agency

#### 3.2.1. Semantics

Historically, semantic information was contrasted with syntactic information. Syntactic information quantifies the kinds of statistical correlations between two systems without giving meaning to those correlations (Kolchinsky and Wolpert, 2018). This is used predominantly with Shannon's information theory, which is a measure of the reduction of statistical uncertainty between two system states which can differ in time.

Some studies (going forward known as the study or this study) have distinguished between syntactic and semantic information in systems (Kolchinsky and Wolpert, 2018). This study attempted to create a formal definition of semantic information that is applied to both “living” and “non-living” beings (any physical system like a rock or cell, for example). Herein, semantic information was defined as *information that a physical system has about its environment which is causally necessary to maintain its existence over time*. The qualitative aspect of semantic information is related to the intrinsic dynamics of systems and their “environments”. The *quantitative tools* used to calculate semantics are information theory and non-equilibrium statistical mechanics.

Importantly, the study is distinguished between “meaningful bits” and “meaningless bits”. This also allowed for a differentiation between sub-concepts of semantic information such as “value of information”, “semantic content”, and “agency.” Semantic information then is defined as information that enables systems to achieve their goals (maintaining a low entropy state). However, this is not an exogenous (goal derived from or measured from “external” sources) approach. Any “meaning” obtained from exogenous studies is meaningful (in terms of goals) from the *perspective of the observer* or scientist, and *not the system itself*. The difference in this study as compared to others is that the others offer standard teleo-semantic approaches where goals are understood in terms of evolutionary successes such as fitness. These standard approaches are suited more for systems that change according to selection; they do not describe systems that are “non-living” or synthetic (Kolchinsky and Wolpert, 2018). They also tend to be etiological, in that they are based on past histories of the system. The approach presented in this study instead creates an account of semantic information based solely on the intrinsic dynamics of a system in an environment without regard to its past or origin. Therefore, it presents an attractive model for an account of agency which includes AI systems. This is an autonomous agent model which requires that a not-in-equilibrium agent maintain its own self-existence/maintenance in an environment. This is active self-maintenance where agents use information about the environments to achieve their goals, and hence this information is intrinsically meaningful for them (Kolchinsky and Wolpert, 2018). This kind of perspective also *applies to robots and “non-living” systems*. This intrinsic goal is neither obtained from an exogenous source nor is it based on past histories or origins. Importantly, semantic information is derived from the *mutual information* between the system and its environment (within the initial distribution, which is defined as stored semantic information).

#### 3.2.2. Viability and value

The study coins the term “viability function”. Viability functions are used to statistically quantify the system's degrees of existence at any given time (hence, one can say that viability functions describe real-value aspects of systems). For this, a negative Shannon entropy is used (it provides an upper bound on the probability that the system occupies any small set of viable states). Semantic information now means the information exchanged between the system and its environment which causally contributes to the system's existence. It is measured by the maintenance of the *value of the viability function*. To quantify causal contributions, the study used a counterfactual intervened distribution in which there was a *scrambling of syntactic information* between the system and its environment. The value of information was defined as the difference between the system's viability in time after the intervention. A positive difference would mean that there was some syntactic information between the system and its environment which plays a causal role in maintaining its existence. A negative difference would mean that the syntactic information would decrease the system's ability to exist.

To describe the value of information, the study gives the example of a rock. A rock has a very low dynamic and thus it can remain in a low entropy state for longer periods. If information is then scrambled by swopping rocks from their current environment into different ones, this intervention would not make much difference to the rock. However, by doing the same thing with a hurricane (my modified explanation) that requires specific conditions for its maintenance, the result is that the hurricane has a greater set of parameters for its maintenance. If those parameters are not met, it will dissipate (viability decreased)—and thus it has some important semantic information. Therefore, the semantic information is important for hurricanes, and this would likely be greater for hurricanes than rocks. If you put an organism in a new environment it may not be able to find its own food, hence organisms place a higher value on information.

#### 3.2.3. Viability, syntactic, and semantic information

Non-equilibrium systems are those in which the non-equilibrium status is maintained by the ongoing exchange of information by sub-systems. An example of this is the “feedback-control” process in which one subsystem acquires information about another subsystem and then uses this information to apply controls to keep itself or the other system out of equilibrium (like Maxwell's demon). Information-powered non-equilibrium states differ from the traditional non-equilibrium systems considered in statistical physics which are driven by work reservoirs with control protocols, or which are coupled to thermodynamic reservoirs (Kolchinsky and Wolpert, 2018). The reduction of entropy thus carries costs in the expenditure of energy as heat. Within the thermodynamics of information, Launderer's principle states that any process that reduces a system's entropy (by x number of bits) must release energy in the form of heat. Heat generation is also necessary for the acquisition of syntactic information. *Viability* is connected to this reduction of entropy through semantic information acquisition. Semantic efficiency in the study speaks to a quantification value of how much the system is “tuned” to possess only syntactic information which is relevant for maintaining its own existence. The semantic efficiency is related to the thermodynamic multiplier which is the measure of the “bang-for-buck” of information (below). This simply asks, “what types of information would carry more benefit than other types?” Systems with positive values of information and higher semantic efficiency tend to have a larger thermodynamic multiplier (Kolchinsky and Wolpert, 2018). Stored semantic information is not that which is acquired during dynamic exchanges with environments. Rather, it is the *mutual information* between systems and environments that is also *causally responsible for maintaining viability*. It is important to note that systems with low entropy are not the same as remaining within a specific viability set. This means that systems do not need to maintain the same “identity” over time to maintain a low entropy state. Identities can change while still maintaining low entropy states. Hence, a specific identity profile (like a human) is unnecessary for an account of agency.

Observed semantic information in the study speaks to that which is affected by the dynamic interventions that scramble the transfer of entropy from the environment to the agent. This kind of information identifies semantic information which is acquired by dynamic interactions between systems and environments (not mutual or stored information). The syntactic information in the study is scrambled to obtain semantic information. This is how *meaningless* and *meaningful* are obtained (optimal intervention determines this). Any information that can be scrambled without affecting viability is meaningless and that which must be preserved to preserve viability would be meaningful. Both observed and stored information are necessary for viability preservation; however, observed speaks to dynamic interactions between systems and their environments. The semantic efficiency ratio is the ratio of the stored semantic information to the overall syntactic information (Kolchinsky and Wolpert, 2018).

Systems can have a non-unique optimal intervention, namely, *multiple variable and redundant sources of semantic information* which are used to maintain viability (like relating to different food sources, see Kolchinsky and Wolpert, 2018). This is important when considering the different dimensions of society in which systems are integrated. Relevant reservoirs depending on the system, its context, and its function can include sexual reservoirs, ethical/behavioral reservoirs, different knowledge domain reservoirs, socio-political reservoirs, and socio-emotional reservoirs. This presents a paradigm and mechanism to determine the status and inclusion of certain systems in certain contexts by assessing their suitability to participate adequately in that context. The thermodynamic multiplier provides a means to determine *suitability*.

#### 3.2.4. The thermodynamic multiplier

The thermodynamic multiplier is the stored semantic information (the benefit–cost ratio of mutual information) that provides a manner of comparison for the ability of different systems to use the information to maintain their viability (Kolchinsky and Wolpert, 2018). This would mean that the stored semantic information gains its status *based on its benefit outweighing its cost*. If the information value is positive, then having a low semantic efficiency means that there would also be a low thermodynamic multiplier. Therefore, “paying attention to the right information” in terms of semantic efficiency is also correlated with thermodynamical efficiency. It is a measure of the thermodynamic costs of obtaining new mutual information compared to the viability benefit obtained from that acquisition.

#### 3.2.5. Transfer entropy and semantic efficiency

Observed semantic information can be acquired in dynamic interactions through the use of transfer entropy. This is a measure of information flow and is widely used and understood. The transfer entropy movement from the environment to the system is not necessarily the same as the flow from the system to the environment. Observed semantic information describes dynamic actions and decisions where any information scrambling that comes from the environment to the organism *would result in an impact on viability*. For example, Jack and Jill went up the hill with Jack leaving behind a trail of breadcrumbs to lead them back home. If at some point during their adventure, a wind were to blow away those breadcrumbs then they would not know their way home, affecting their ability to survive or feed themselves. The transfer entropy would speak to the breadcrumbs which would have observed semantic information because the breadcrumbs as an object contain an informational interaction between a system (as agent) and the environment. Thus, the *value of the transfer entropy* is the viability value at a specific time before scrambling versus the viability value after scrambling. This value is then known as *semantic efficiency* (Kolchinsky and Wolpert, 2018).

#### 3.2.6. The agent

An autonomous agent (and autonomous agency) in this system would be a physical system that has a large measure of semantic information (Kolchinsky and Wolpert, 2018). One can identify autonomous agents by finding timescales and system/environment decompositions which maximize measures of semantic information. This would in turn depend on the thermodynamic multipliers, the transfer entropy, and the amounts of semantic information. It is, however, important to remember that semantic information can have a negative viability value. This means that it can be mistaken/misrepresented information that is used in a way that harms the agent's viability value. The study also highlights that semantic information requires an *asymmetrical measure* (unlike syntactic mutual information). This is because this information concerns the *viability of the system* and not the environment. This system also does not require the decomposition into separate degrees of freedom (such as sensors, effectors, membranes, interior, exterior, brain, or body). Thus, it is not about internal representations *but rather about the intrinsic dynamics* of the system and its environment. This can also be used to create an account for “life”.

## 4. Conclusion

My methodology, using successful observable, predictable experiments that provide more information, is more accurate and enables a method of grading or ranking systems as agents according to domain suitability. This relies on the use of semantic information and its relationship with viability. To summarize, viability (reducing or maintaining a low entropy state) is the ability of a system to continue to exist, and it is measured in terms of the viability function. Changes in this viability function are determined by counterfactual dependences obtained through the scrambling of syntactic information. This enables the ascertainment of the more “valuable” semantic information as causally contributing to the system's viability function. There are two kinds of semantic information, both of which affect the viability function: (1) stored and (2) observed. Stored semantic information is the *mutual information* between systems and environments, while observed information is that which is acquired by dynamic exchanges between systems and environments. One can obtain observed semantic information by scrambling the transfer entropy. The observed semantic information is necessary to determine actions and agency since it describes dynamic “active” interactions. Furthermore, survival in this instance is de-linked from “biological” systems and is measured according to maintaining a system's viability based on its own intrinsic dynamics. This presents an attractive way to create a general and invariant account of personhood and agency. I also presented an account of what constitutes rarity. This provides a further attractive way to grade “emergent” information content or properties.

This account, routed in Kantianism, recognizes the explanation and information issues in alternative accounts and provides a more accurate framework. Legal systems and ethics discourse should take note of this account as the usual ways in which these conversations are entertained and are doomed since they tend to rely on poorly understood, ephemeral notions such as “consciousness”. Instead, systems should be evaluated according to their own intrinsic properties which enable a better approach to determining suitability (agency and personhood) because it considers agents as agents within their own informational paradigm and not relative to another agent's informational paradigm. In this way, intrinsic bias is made to be a strength when it is considered from the perspective of the system itself.

## Data availability statement

Publicly available datasets were analyzed in this study. This data can be found here: https://osf.io/evna6.

## Author contributions

MN: Conceptualization, Investigation, Methodology, Project administration, Resources, Visualization, Writing—original draft.
