# Supplementary material for: What does it mean to be an agent?
Source: Front Psychol. 2023 Oct 17;14:1273470. doi: 10.3389/fpsyg.2023.1273470 (PMC10616898; doi:10.3389/fpsyg.2023.1273470)
Supplement: Supplementary file 1 [file Data_Sheet_1.PDF]

Supplementary Material

What does it mean to be an Agent?

Meshandren Naidoo*

*** Correspondence:** Meshandren Naidoo: 214549331@stu.ukzn.ac.za

1 Part A

1.1 Explanations

Explanations are like proofs of logic and are usually derived or deduced from a defined set of axioms. However, what explains those axioms is separate from the deductions that are based on a system of them. Thus, explanations constitute the deductions themselves (the explanadum), and potentially also their sequence of flow. People often rely on explanations without being able to explain why they do so (Keil, 2006). When explanations are presented in a way that fails to offer a mechanistic flow of events, they are often understood as "effects" instead of explanations. Within the scientific paradigm, explanations must have predictive value. Explanatory understanding consists of creating, discovering, providing and receiving explanations (Keil, 2006).

1.2 Counterfactual Example and Explanatory Loss/Unsuitability

An example of building off the description of counterfactual follows: Consider a cannonball (B1) with a mass of one kilogram (M1), which is fired at a glass pane that does not break. Then consider another cannonball (B2) with a mass of two kilograms (M2). This is fired at the glass pane, which then breaks. Both cannonballs are fired at the same velocity. What is the difference that causes the shattering of the glass pane? The method of difference would suggest that the masses (since there is difference) should have ontological superiority (and privilege in explanations) as the cause of the shattering. However, this is incorrect; it is the *momentum* that is the cause. One cannot decompose momentum into mass and velocity logically to privilege one over the other. As mass and velocity are multiplied to get the resultant momentum one cannot then attribute mass as having the ontological superior status in causal outcomes. The difference in mass is the cause of (B2)'s capability to shatter the glass pane (by affecting momentum), but it is not the cause of the shattering itself. Therefore, the method of difference gives us *less general information—and is not accurate*. Mill (1843) recognized that this approach made metaphysical assumptions, namely that causes compose in orderly ways which allow for decomposition.

1.3 Mutual Information and the Kelly Criterion

Kelly developed the Kelly criterion which demonstrated that when gambling or investing in situations where there are repeated events, the mutual information $I(X;Y) = H(X|Y)−H(X)$ would characterize the increase in expected growth rate of wealth when side information $y \in Y$, which is related to event outcomes, is available. As Taleb touted, Kelly was simply asking how much one could rely or bet on the value of one variable if the value of another variable is known. If the value of variable (1) is certain

to be obtained, the mutual information would be one (1). If the certainty of obtaining the value of variable (1) is only 50% or a one-half, then the mutual information would be closer to zero (0) than being halfway between zero (0) and one (1).

1.4 Laws

Notions of law include exceptionless generalizations which are: (1) representable by universally quantified conditionals; (2) contain only qualitative predicates; (3) non-referential to particulars like objects or spatio-temporal locations; (4) widely applicable; (5) supportive of counterfactuals; (6) confirmable or predictable; and (7) integrable into systemic theories or to unifying other laws or inquiries (Woodward, 2000). Most of these criteria do not assist with particularity, specifics, detail, or understanding features of laws themselves. Explanatory status depends on the range of interventions and changes over which a generalisation is invariant. This is what also determines whether something qualifies as a law. Traditionally, there is a dichotomy operating which states that either a generalization is a law or, if not, then it is accidental or a matter of chance (Woodward, 2000). This assumes that the boundary between laws and non-laws coincides with the boundary between explanatory generalizations and non-explanatory generalizations (Woodward, 2000). First, invariance coincides with an account of autonomy; the more invariant a generalization is (as above), the greater the degree of autonomy it has (Woodward, 2000). That is also what it means to be robust or antifragile. Whereas laws do not properly account for the need for symmetries, invariance highlights the need for symmetries in explanations and provides a method (Woodward, 2000). The failures of invariance are also massively important. Among other things, invariance allows for the construction of an observable theory of agency and hence a higher quality explanation. Furthermore, it is also important in the consideration of the teleological purposiveness account that I introduce. Invariance provides a means to highlight the informational insufficiency of the notion of “chance”.

2 Part B

In the *Critique of Pure Reason* (Kant, 1998) and the *Critique of Judgment* (Kant and Bernard, 1790), Kant offers an understanding of how humans construct meaning. What he explores is the meaning of “subjective” and “objective” as a concept and, in a way, the meaning of concept itself—the concept of “concept” as it were. A ‘concept’ is a basis to understanding processes. Later biological philosophers such as Johann Wolfgang von Goethe (Abrams, 1953) adapted this term into the realm of genius and creativity.

Kantian judgement is based on the Aristotelian idea of forms and unity. Biological beings have an inherent end (form) into which they develop (Abrams, 1953). The process by which they achieve this end is mechanical, thus falling within the *phenomenal world*. Biological beings are not a combination of parts of prior design or composition. Kant refers to the moving power and the formative power (Abrams, 1953). Kant then presents a conceptual analysis involving a tree to illustrate this point.

A tree’s genesis is another tree. That tree has an inherent form, which is the formative power, and the moving power (Abrams, 1953) is the ability of the tree to develop from within through the assimilation and transmutation of the external into something useable and into itself. The conceptual analysis speaks to parts and wholes. Parts are requisite to form wholes. Parts are characterized as such because of their relation to the whole. The whole, on the other hand, is equally characterized as such because of its parts. Thus, each gets its content or existence from the other. In other words,

meaning is constructed through the mutual dependence of antithesis. To link this back to the discussion of creativity, creation gets its meaning from that which already exists. Creativity means to go from non-existence to existence. Thus, the process of creativity requires a mutual dependence of the already existing and the non-existent.

Back to the tree, the parts of the tree develop to form the sum, being the whole, and the whole is the sum of its parts. There is an interdependence between the parts and the whole—the concept of the whole needs the concept of the parts, and the concept of the parts requires the concept of the whole. The tree, and other biological organisms, are thus unconsciously teleological (Abrams, 1953). The whole and the parts as concepts are thus both means and ends. In doing so, Kant also partly solves the conundrum of how genius can exist without prior knowledge or consciousness.

Gerd Sommerhoff (1950) alludes to how the distinguishing capacities of organisms also serve as a criterion of purposiveness:

> "On the phenomenal level from which all science must proceed, life is nothing if not just this manifestation of apparent purposiveness and organic order in material systems. In the last analysis, the beast is not distinguishable from its dung save by the end-serving and integrating activities which unite it into an ordered, self-regulating, and single whole, and impart to the individual whole that unique independence from the vicissitudes of the environment and that unique power to hold its own by making internal adjustments, which all living organisms possess in some degree".

3 Part C

In *How Humans Judge Machines*, Hidalgo (2021) summarizes the results of multiple experiments with nearly 6,000 participants (in the US alone). These experiments were designed to compare human perceptions on machines and those on humans in the same, or very similar, situations. Hidalgo (2021) concludes that people judge humans by their intentions, and machines by their *outcomes*. In relation to moral judgements (based on moral agency, autonomy, and moral status) the study found the following:

- To make a moral judgement one must grasp concepts like harm, fairness, loyalty, authority, and purity.
- Moral judgements are based on emotion, intuition (or automatic association), or social learning (like culture). Reasoning then happens *ex post* as a justification.
- People attribute some moral status to robots, especially where they can express social cues.
- It was also noted that since AI is subservient to goals set by others, it has less moral agency and responsibility. Moral agency is thus seen to lie in *goal making*—rather than executing that goal via action.

The concept of intentions did not develop to describe mental states. It was a manner of explaining actions. Second, given the above, it is possible to construct a non-sentient account of agency. Experimental data in a follow-up article demonstrated that humans' perception of machines aligns closer to their perceptions of humans when they perceive the machines as having *more agency* (Zhang et al., 2022). It was the perception of agency that mattered, and not experience (sentience—

the ability to feel, including pain and emotion). The follow-up study also noted that people aligned machines closer to humans, not in terms of moral status but in terms of intentionality.

There is also a new model for framing agency. The Technological Approach to Mind Everywhere (TAME) approach offers a solution by posing a framework for open embodied cognition (Levin, 2022). Although I cannot here propose a proper account of the framework in its entirety, I highlight the points of salience. First, TAME does not draw rigid lines in the sand regarding what real cognition is, hence making TAME more flexible and accommodating to different kinds of cognition. It also does not discriminate on the substance of the source from which it emanates (cognition does not need to be a biological or human only ability). Furthermore, TAME does not differentiate in terms of the ephemeral and pop-psychology notion of "intentionality". The cognitive account works on a continuum (cognitive meaning information processing ability), from simpler cognition to more complex cognition (such as unlimited associative learning and symbol manipulation). In terms of agency, TAME proposes that this is an empirical question. It proposes that cognition is observer-dependent, empirically testable, and depends on the scientific value of contribution. The appealing aspect of TAME is that it reframes agency, not only as existing on a spectrum, but also in terms of predictability and control. Those systems which display higher empirical validation of including the other axioms (like scientific value) can be construed as having a higher agency. TAME inverts the current thesis on agency into one in which human involvement is a strength rather than a weakness.

4 Part D

The Bell curve (or the Gaussian) is often used for analyses and in the policy-making space as a risk measurement tool. Within the policy space, the misapplication of the Bell curve can be traced back to Sir Francis Galton (Goertzel, n.d.). There is a prevalent misapprehension that science and politics are divorced, yet they are certainly one and the same. Using this method, most of the measurements are around the average and the odds of deviations decline at an exponential rate (rate of decline is increasing) the more one moves away from the average. As one moves away from the average, the odds of getting a piece of information outside the average becomes less and less (almost impossible) (Taleb, 2007). One can then "ignore" outliers because they are so improbable. This is the non-scalable effect of this method. The issue with this is that it is highly fragile and vulnerable when estimating tail events; any small estimation error will result in the predictive capability of this model being completely incorrect and unreliable. Another issue is that of inequality (Taleb, 2007). Inequality decreases as deviations increase(s) because of the increase in the rate of decrease (described above). With scalables, inequalities remain the same throughout. *It paints an inaccurate depiction*.

The Gaussian is attractive because it makes things appear less random, thus giving us the delusion of certainties. *It does this because it averages by using large sample sizes* (like the law of large numbers). As sample sizes increase, the average displays less dispersion and the distribution is narrower (Taleb, 2007). Therefore, uncertainty appears to be reduced, and variations are often referred to as "errors".

Furthermore, standard deviations only exist within Gaussian systems (Taleb, 2007); *standard deviations only get their explanatory power within these systems*. This means that the Gaussian is one of a small class of methods in which standard deviation can be logically used to describe information. *The only area of applicability is qualitative, and not quantitative (yes or no answers, where magnitude does not matter).* There is often a misunderstanding of what standard deviation means (or

sigma or variance). Standard deviation is not the average deviation; *it is a number to which things are scaled to create a correspondence between phenomena as if it was Gaussian* (Taleb, 2007).

Gaussians are the *only* systems in which *correlations and regressions also apply*. Correlations have no explanatory or predictive powers outside of Mediocristan or Gaussian systems. Yet, correlative methods of reasoning are still used to enable decision-making/informing decisions in non-qualitative or Extremistan systems. If one were to analyze the standard deviation within Extremistan systems, one would find that *every sample would have a different standard deviation*. Therefore, it is completely misleading/inaccurate to use standard deviation outside of its small area of application (Taleb, 2007).

5 Part E

5.1 Modern Synthesis

For the past century, evolution was thought to be based on changes in the genes of an organism. This is the Modern Synthesis theory, which views genes as central in evolution (Walsh, 2015). Genes encode instructions for the building of phenotypes, which are combined to form a blueprint for an organism and are passed from parents to offspring (Walsh, 2015).

Modern Synthesis views inheritance, development and adaptions as independent systems with their own causes. While inheritance is the transmission of replicators, development is the expression of the phenotype encoded in replicators (Walsh, 2015). Inheritance and development constitute the conservative aspect of evolution, separate from the creative aspects of evolution—mutation and selection. This separation in causal labor translates to a separation (fractionation) in explanation. Modern Synthesis requires the independence of the underlying processes in inheritance and development. This is because of an assumed asymmetry between them. It is thought that *organisms develop traits which they have inherited, but do not inherit traits which they develop.* Separation and independence are the root of the distinction between *nature* and *nurture.*

Certain unalterable and inevitable traits are inherited from our ancestors. They form the blueprints for building and capacities. Any acquired traits are merely additional to that blueprint and do not play an evolutionary role in lineages.

While both inheritance and development do not introduce adaptive bias (bias in terms of suitability in a niche), selection does. Selection brings about phenotypic changes by choosing among individuals those who are better suited to their conditions. Since selection is distinct from inheritance and development, it requires an independent cause—the external environment. In this view, the agent of selection (the environment) must be independent, autonomous, external, and able to exert unmediated causal influences on organisms (Walsh, 2015). This creates an explanatory distinction—that of internal versus external. While mutation, development and inheritance are internal processes, selection is external as it is imposed by the environment. Thus, constraints are internal, while change or adaptation depend on the environment. Based on this view, the significance of organismal development in evolution is trivial when compared to the other elements as it does not introduce, transmit or choose between evolutionary characters, but rather delivers replicators to the environment. It is this schema that pits “form” against “function” (Amundson, 2005), development

against selection, and the forces of conservatism against the forces of change. This same schema relegates organismal development to a place of minor importance in evolution (Walsh, 2015).

5.2 Situated Darwinism

Unlike Modern Synthesis, Situated Darwinism inverts the explanatory priority above and posits development as being primary in evolution. Development includes any processes which contribute to the formation, maintenance or alteration of individual form, function, or its interactions with its conditions of existence (it is not translation of genetic codes into a phenotype). Development is the source of novelties; it biases evolutionary change and underwrites the transgenerational stability of form necessary for inheritance (Walsh, 2015). Therefore, Situated Darwinism dissolves the Modern Synthesis fractionation distinctions.

5.3 Inheritance and Development

Situated Darwinism both reverses and rearranges the component processes of evolution. In terms of inheritance and development, the view is that replicators are not the sole explanation for inheritance. Inheritance is the intergenerational stability of patterns of similarities and differences within lineages. Offspring should resemble their parents more than others in their lineage. The replication process is vital, but so too are the adaptive responses of the genome, cells, and entire organism. The organism is seen as an adaptive and purposive agent. It transduces, assimilates and harmonizes these causal influences. The result is that it secures the differential resemblances of offspring to their parents. Therefore, intergenerational stability of form is maintained by the robustness of development (Walsh, 2015).

5.4 Collapsing Barriers

Modern Synthesis relies on the Weismann barrier to designate a separation between traits that are evolutionary and traits that are not. However, Situated Darwinism collapses the distinction between evolutionary and non-evolutionary characters, and thus too the Weismann barrier which is predicated on it. In terms of the evolutionary and non-evolutionary characters, there can be no distinction between the two, and any intergenerational trait can be evolutionary. This is because any trait can contribute to individual fitness, and this trait can change in relative frequency over time in predictable ways. Traits too can be different in terms of their robustness or stability within evolution (Walsh, 2015).[1]

The Weismann barrier distinguishes between innate and acquired traits, evolutionary and non-evolutionary characters, and the inheritable versus the non-inheritable, and is central to Modern Synthesis. The Weismann barrier is a result of the idea that changes downstream of DNA via developmental systems do not readily reflect on DNA, and is the basis for the primacy of genes (replicators) in evolutionary explanation (Walsh, 2015). However, Situated Darwinism negates the Weismann barrier. Even if changes in downstream processes do not affect the DNA structure, they can impact function (Walsh, 2014). The function of germline entities are context-dependent. DNA is continuously altered and fixed and cells change a genome’s structure and function (Walsh, 2015), so

[1] This is because any trait can contribute to individual fitness, and this trait can change in relative frequency over time in predictable ways.

making it apparent that genomes are "read-write", and not read-only, memory systems (Noble, 2013; Shapiro, 2011).

5.5 Fit and Diversity

Explanations of things should center on what is, why it is, what is not, and why it is not. The "what" speaks to functions; the "what is not" speaks to constraints. Modern biology speaks to fit and diversity of forms as both functions and constraints. Modern Synthesis views natural selection as the singular source of adaptive biases in evolutionary change (thus explaining function). When selection promotes fit, it also causes populations to split and, in this way, it promotes diversity. However, natural selection does not explain the divergences of biological forms—*because the bearers of these forms are whole organisms*. Each organism is subjected to environmental conditions and is not simply an assembly of individual traits. The Whole (not the parts) is what is subject to, and creates, activity. At each stage of development, Wholes have functions—including eggs, for example. Development is limited to the resources available to an organism at the time. This means that not everything that can or may develop, actually develops. Therefore, the explanation of form can be accounted for in terms of selection and development. Modern Synthesis argues that selection is the adaptive mechanism—it explains why something is (for functional traits). Development, on the other hand, is conservative and explains why it is not.

5.6 Natural Selection and Development

The classical approach is the two-force model, which stipulates that natural selection and development are distinct and often compete. Natural selection is thought to be a source of creativity and elimination. Selection is viewed as acting systematically to enhance complex systems through the genotype; it functions as a negative force (constraint) and a positive force in that it pays a premium for contributions to improvement (Walsh, 2015). This has led some evolutionary thinkers to view selection as "creative" (Mayr, 1976).[2] Selection acts like an evolutionary sieve in which beneficial mutations are kept and harmful ones are discarded. It can create novelty through increasing the chance of genetic combinations which would otherwise be unlikely (Ayala, 1970). The argument is that genotypes create phenotypes, and phenotypes in these environments are subject to selection. Development is seen as a contributor to evolution, but only in the sense that it constrains (Maynard Smith et al., 1985; Walsh, 2015; Wagner and Altenberg, 1996)[3] or biases the range of possible forms which selection can then affect (Maynard Smith et al., 1985). Adaptations then evolve in this constrained space (Wagner and Altenberg, 1996).

The nature of the developmental system that exists constrains or channels change of form that is acceptable, so limiting selection in what it is able to achieve, given some starting anatomy (Raff, 1996). Ontogeny or development can explain only why something is not (in relation to what it is). This conception of development flows from the two-force model. Both selection and development have influence in the distribution of biological form, but only one of them promotes adaptive evolutionary change. In addition, this approach also divorces constraint from natural selection—with

---

[2] These include Theodosius Dobzhansky, Julian Huxley, and G.G. Simpson.

[3] For example, John Maynard Smith expresses this common definition of development as a constraint: "A developmental constraint is a bias in the production of variant phenotypes or a limit on phenotypic variability caused by the structure, character, composition, or dynamics of the developmental system". Adaptive evolution is explained by topographies—namely that it takes place on an adaptive landscape. "For instance, developmental constraints frustrate selection by restricting the phenotypic variation selection has to act upon. Adaptations would be able to evolve only to optima within the constrained space of variability."

the result being that there is a dichotomy and quest for dominance between selection or constraint (like genetics versus development). These are false dichotomies.

Stephen Gould argues that evolution should largely be understood as the study of how laws of form manifested in development affect diversity. Gould contends that the principles governing the development of form, historical contingency and selection are just as important in evolutionary theory. However, Modern Synthesis states that development only contributes to fit a diversity of form by constraint, and therefore natural selection must take priority.

The two-force model has two fundamental flaws. One is empirical and the other is conceptual. The former is that development does not just constrain adaptive evolution, and development makes a systematic contribution to the adaptiveness of evolution. Furthermore, at a population level, selection is a higher-order effect, and not a population-level cause.

5.7 Development and Adaptiveness

Evolutionary development biology strives to give development a more prominent role. The field has produced research which demonstrates the productive role of development in adaptive evolution. Important in adaptive evolution is "regulatory evolution"—the position that the genomes of organisms contain toolkits of regulatory genes that control development by influencing timing or products of genetic expression of other genes. These regulatory genes and their processes can direct the development of similar phenotypes despite differences in the underlying developing tissues and variations in the structural genes. Changes in regulatory roles of genes can also produce important changes in the structure and function of phenotypes whose development they regulate. "Facilitated variation" highlights the importance of regulatory evolution for adaptive change. Organisms' genomes consist of a core of conserved components: these are highly regular structures shared across organisms. These core components underlie the production of numerous different phenotypic structures, both within organisms and between lineages. These highly conserved core structures and processes combine with the more variable developmental resources in producing novel phenotypes. The preserved core variable periphery structure of developmental systems gives organisms their dynamic robustness and the ability to maintain viability by making compensatory changes to perturbations. It also allows development to "search" the morphological space and find new stable and adaptive structures. Thus, development is important in promoting adaptive evolution.

Evolutionary creativity does not rely only on selection. Through its ancient repertoire of core processes, an animal's current phenotype determines the type, amount, and viability of phenotypic variation that can be produced (Gerhard and Kirschner, 2007). Regulatory circuits can be used for new roles. This allows for genes to be used in novel structures with only a small number of regulatory changes. Evolutionary changes in gene regulation produce diversification of novel characters. New patterns can emerge from regulatory modification of genetic interactions in development—within and between species (Carroll et al., 2000). The re-deployment of old regulatory resources in novel ways is arguably the main mode of evolutionary and adaptive change. Most evolutionary changes have come from regulatory changes instead of changes in core processes, since the Cambrian era (Walsh, 2015). These regulatory changes alter how core processes are

used/activated; any new manners of usage include amounts, timing, combinations, and instances of activation (Kirschner and Gerhard, 2005).

Development is a dynamic and robust process, and gives organisms the capacity to innovate. Through its phenotypic repertoire and the phenotypic accommodation to new forms, developmental processes can produce new and stable forms without the need for genetic mutations (Moczek et al., 2011; West-Eberhard, 2003). Therefore, evolutionary changes play roles in gene regulation.

Modularity allows developmental systems to do this. Developmental modules are tight and integrated internal units. Developmental modules have weak regulatory interactions with each other, and this is the general feature found in all adaptive and self-organizing systems. Modular systems allow for many different and distinct phenotypes to be created through re-organizing internal module connections—including the "relatively" independent evolution of each module itself (Kitano, 2004). This tight integration and the strong decoupling with others allows two important features of developmental dynamics. First, robustness of each module is capable because of the internal integrations: they can compensate for perturbations. Second, decoupling protects modules from potentially disruptive changes which may happen in other modules. Each module is usually able to produce many stable outputs, but this depends on the context that the module is operating in. Thus, module architecture allows organisms to robustly and reliably produce viable organisms of its kind, and to generate phenotypic novelties as an adaptive response to genetic, epigenetic, or environmental perturbations.

Adaptive plasticity is also fundamental for adaptive evolution (Pfennig et al., 2010). Phenotypic plasticity enables development processes to respond to their contexts and environments. The source of novel phenotypes is rooted in responsive phenotype structures, and it is irrelevant in terms of development whether the recurrent change (novelty) is caused by mutation or environmental factors (West-Eberhard, 2003). Wagner (2011; 2012; 2014) highlights how phenotypic robustness drives adaptive phenotypic evolution, and how development systematically contributes to adaptive bias in evolution. Neutral networks have high adaption-promoting properties—they can act as evolutionary capacitors. Neutral networks for different phenotypes overlap in gene space, and thus different phenotypes can be produced by the same or similar gene networks. One neutral network can overlap with others. This allows for development to "search" the space of viable phenotypes efficiently. Thus, the robustness of development (because of the architecture of gene networks) contributes to adaptive evolution in two ways: (1) robustness causes the existence of genotype networks—complex web-like structures formed by genotypes with the same phenotype, which facilitates phenotypic variability; and (2) a robust phenotype can help the evolutionary exploration of new phenotypes by accelerating the dynamics of change in an evolving population. Therefore, development is fundamental to evolutionary adaptation.

Evo-devo (Hall, 2012) argues that genomes' variational capacities are the functions of developmental systems in which they are embedded—namely, through modular organization, dynamics of their mechanistic interactions, and their non-programmed physical properties (Müller, 2007). Development can contribute to adaptive evolution in three ways (Hendrikse et al., 2007; Pfennig et al., 2010): (1) it biases the direction of variation through adaptive novelties; (2) it controls the amount of phenotypic variation by buffering development against mutations and their effects;[4] and (3) it is a

[4] This secures the viable production of phenotypes.

capacitor for storing latent variation, and a phenotypic repertoire which can influence the direction and rate of future evolutionary change (Walsh, 2015).

Therefore, development (and not natural selection) may be the source of adaptation. While Modern Synthesis focuses on explaining adaptive changes as population dynamic events, evo-devo theory explains phenotypic changes through adjustments in developmental systems (including physical interactions between genes, cells, and tissues). This explanation is indifferent to whether the change is adaptive or not (Müller, 2007). Therefore, we must also consider development as a source of adaptive bias in evolutionary change. It is inaccurate to think of development as merely a constraint on adaptive evolutionary change—Modern Synthesis states that only selection causes adaptively biased evolutionary change. Evo-devo supporters note how this shift towards including developmental systems in evolutionary theory must result in a shift of explanatory emphasis on external factors of natural selection to internal dynamics of the said systems (Müller and Newman, 2005).

5.8 Selection

Modern Synthesis mistakenly pits selection against development because it views selection and development as independently responsible for changes in population structure. However, selection is a higher-order effect, which reflects the high level of population structure of the aggregate of several causes of individual-level births, deaths, and reproductions. Selection is thought to be spontaneous—not requiring any new additives to individual-level causes (birth, survival, reproduction, and death) for populations to undergo selection-orientated changes. Selection as a population cause and the individual-level causes would result in too many causes making selection causally redundant (Brunnander, 2007). However, this does not mean that selection is *explanatorily redundant*.

The relevant type of explanation for population dynamics is known as "higher-order effect explanation". These explain how ensemble-level trends/patterns appear from many complex and disordered activities within ensembles (like populations undergoing adaptive change) (Walsh, 2015).[5] Therefore, natural selection and development are *related but not opposing*—they are parts to a whole. Selection at population level is composed of development at the individual level. Selection is the higher-order effect of individual level causes of population change. Development is the individual-level cause of population dynamics. They are not independent of each other. Darwin discovered that individual organism activity is adequate to explain fit and diversity. Every cause of adaptive evolution can be found at the individual level of organismal development. Therefore, all causes of bias in evolution are from development. This is the conceptual error of the two-force image. If adaptive evolution is development, it is a mistake to view development as being principally against adaptive evolution (Walsh, 2015).

5.9 Causes of Adaptation

Modern Synthesis posits that adaptive evolution arises through the accretion of small, rare, independent, and beneficial mutations. Selection then eliminates the deleterious mutations, retaining and combining the good mutations, which results in a well adapted organism. However, this does not explain the conditions which need to be satisfied for mutations to accumulate. How can systems like

[5] For example, how gases move towards thermodynamic equilibrium or the process of diffusion.

this emerge through random mutation and selection? How can they accumulate useful mutations? (Kauffman, 1993).

Modern Synthesis locates the sources of adaptation in the intrinsic property of genes. Modern Synthesis argues that genes are replicators: independently acting units of small phenotypic effects whose activities are reasonably constant across contexts. Therefore, the behavior of genes affects the adaptiveness of organisms. However, the notion of "evolvability" asks which features organisms must process in order for their lineages to evolve. The importance of evolvability is evidenced by the fact that some lineages or organisms are more susceptible to adaptive evolution than others. This hints at the notion that the dynamics of individual development may facilitate adaptive diversity. But development does more than this—certain features of development are prerequisites for the adaptiveness of adaptive evolution. Systems that can accumulate useful mutations are those which can balance two antagonistic demands: stability and mutability.

Schwenk and Wagner (2004) highlight the paradoxical requirements of evolvability. Phenotypes must be mutable and responsive to constantly changing environmental demands. However, phenotypes must also be stable so as not to upset the complex dynamics of their developmental and functional systems (Schwenk and Wagner, 2004). The importance of the conditions that promote evolvability were clarified when programmers used the principles of evolution to improve computer programmes through the introduction of random mutations to the code, and thereafter choosing the best programme. However, only certain computer programmes can evolve in this way. Most crash when random mutations are introduced. For programmes to undergo adaptive improvement, they require special architectural features (Wagner and Altenberg, 1996). A programme must be structured in such a way that most changes do not negatively affect it, but that minimal changes move it to a new and stable state. The key to this capacity is "modular architecture". As with organismal development, the computer programme must be modular. Systems with this architecture show the same robustness as organisms. This suggests that the ability of a population to undergo adaptive evolution is grounded in the capacities of organisms that are manifested in their development: modularity, plasticity, robustness, and innovation. In addition, each of these capacities is a type of developmental constraint (the structure, character, composition, and dynamics of the system) (Maynard Smith et al., 1985). Therefore, the Modern Synthesis view of the role of constraint in adaptive evolution seems to be incorrect. Developmental limitations on individual organisms do not hinder adaptive evolution; they are the main causes of adaptive evolution.

5.10 Complementarity

The competition between selection and development in the Modern Synthesis two-force model is based on the idea that both explanations are mechanistic. Although selection and development may seem to be oppositional, they are complementary.

5.11 Diffusion

As arrangement and distribution are applicable to the same event, they are complementary. The commonality between individual-level and ensemble-level explanations is reflected in evolution. Developmental explanations describe how individual-level causes which function within ensembles (systems) conspire to produce the higher order effect of selection. Distributional explanations (as natural selection) describe how distributional properties of the ensemble (or whole) at a specific time

are a function of their distributional properties at a previous time. Therefore, these are non-competing, complementary explanations for the same phenomena (Walsh, 2015).

5.12 The Two-Level Model

The two-level model should replace the two-force model. Explaining adaptive evolution, fit, and diversity involves two distinct projects: (1) explaining changes in population structure; and (2) explaining the adaptedness of individuals. Natural selection achieves (1). Populations change because of their structure, the distribution of individual-level causes of living, dying, and reproducing. But the two-force model fails to explain the properties of individual organisms, and hence fails (2). (2) requires an explanation of the processes that occur within individuals which predispose them to preserve and initiate adaptively advantageous phenotypes (Walsh, 2015).

6 Part F

6.1 Adaptationism

The adaptationism notion derives from Modern Synthesis. As already alluded to, when none of the processes occurring within organisms introduce an adaptive bias to form, something else must. This something else is natural selection; it shapes form to meet its contextual challenges. This account of adaption is based on two considerations: (1) the autonomy of the environment; and (2) the explanatory externalism paradigm.

6.2 Environmental Autonomy

The environmental autonomy argument is that the environment determines which organisms survive and reproduce by processes of promotion and elimination, thus changing biological form. The result is that the population contains individuals best suited to survive in it. Traits are understood to be units of adaptions; they are responses to pressures on form which come from the environment (Godfrey-Smith, 2001a).[6]

"Niche" describes the relationship between organisms and environments; it speaks to the properties of an environment which organismal forms may or may not fit. *The properties* of the niche are independent of the inhabiting organisms. The issue with this is that it posits that the external world produces problems for organisms which they must solve through natural selection. Therefore, adaption presupposes a pre-existing world which poses problems that adaptation can also solve. There is then a separation between form and niches, and they are asymmetrically dependent. Organismal form depends on niches, but niches do not depend on organismal form (Lewontin, 1978).

6.3 Explanatory Externalism

This adaptationist notion gives explanatory primacy to adaption, promoting influences of external environments over the adaptive neutral process of development and inheritance. This requires us to separate contributions of the environment to change (selection) from those which are internal to biological form (inheritance, development, and mutation). The adaptationist programme is thus

[6] "[O]rganisms respond to the environment, but the environment is largely autonomous with respect to the organisms. The environment is seen as either stable (as far as the time scale of the evolutionary process in question is concerned) or else as changing according to its own intrinsic dynamics".

committed to "explanatory externalism" (Godfrey-Smith, 1996). This decoupling is based on the same assumptions which caused the fractionation of evolution. The processes that introduce adaptive bias in evolution are distinct and independent of processes within organisms, and it is quantitative and qualitative inferences regarding contributions of the environment that point to this. This notion creates the idea of evolution happening in two autonomous domains: the inner realm of the replicator (where they compete, form alliances, and implement their developmental programmes) and the outer realm of the niche/environment (which selects and moulds form). It is only selection which fits organisms to their environments, and organisms are merely bystanders. According to this view, the organism is just a passive object on which evolutionary forces act. These independent forces can be external or internal; one generates problems (at random) and the other generates random solutions (Lewontin, 2001a).

6.4 Organisms and Their Environments

The decoupling of organisms and their environment does not explain the adaptedness of form. The organism itself does. Adaptions are not different responses to the environment per se, *but are rather responses to the organismal experiences of an environment*. This experienced environment is neither external to the organism, nor is it autonomous—it is comprised of the properties of the environment and the way in which the organism experiences and is affected by those features. This depends on the capacities of organisms. Therefore, the Modern Synthesis notion that adaptive evolution requires an external environment to shape form is wrong. Organisms are neither passive nor separate to their conditions under which they evolve,[7] and partly constitute the environments in which they evolve. There is not an environment without an organism and vice versa (they are non-separable and imply one another) (Waddington, 1957; Lewontin, 1978; Gibson, 1979).[8] The environment (as physical), as a perspective of the organism, also evolves. Waddington (1957) realised this. Lewontin (1978) and Gibson (1979) also discussed the notion of conjoining organism and environment.

6.5 Forms and Affordances

Organisms' adaptive activities partially constitute their experienced environments. Organisms are reactive and are purposive entities that make and regulate their conditions of existence. Therefore, the conditions in which form evolves are dialectic. Affordances are relational and dialectical. Gibson (1979) conceptualizes affordances:

[7] Here Gould and Lewontin's call to incorporate organisms "with all their recalcitrant ... complexity" is pivotal. The general idea is that we should resist thinking of organisms as somehow separate from and passive with regard to the conditions under which they evolve.

[8] In realizing this, as in so much else, Waddington (1957) seems to have been ahead of his time: "[O]rganism and environment are not two separable things, each having its own characteristic in its own right, which come together with as little essential inter-relation as a sieve and a shovel of pebbles thrown onto it." Lewontin (1978) said "There is no organism without an environment, but there is no environment without an organism. There is a physical world outside of organisms and that world undergoes certain transformations that are autonomous ... But the physical world is not an environment, only the circumstances from which environments can be made." Gibson (1979) said: "[I]t is often neglected that the words animal and environment make an inseparable pair. Each term implies the other. No animal could exist without an environment surrounding it. Equally, although not so obviously, an environment implies an animal (or at least an organism) to be surrounded ... the environment is ambient for a living object in a different way from the way that a set of objects is ambient for a physical object."

> "The affordances of the environment are what it offers the animal, what it *provides* or *furnishes*, for good or ill ... I mean by it something that refers to both the environment and the animal ... It implies the complementarity of the animal and the environment."

Adaptation is not a response to an environment, *but to an affordance*. Affordance can be understood as an emergent property of the organism–environment system (Chemero, 2003; Stoffregen, 2003). Affordances imply purposive systems, and vice versa. For systems to respond to affordances as affordances, they must be capable of responding to them as such by exploiting opportunities for attaining goals or mitigating impediments. A purposive system is one that is capable of responding to its conditions in ways that are conducive to the fulfilment or amelioration of the impediments to those goals. Affordances are properties of organism/environment systems that have "meaning" or significance for the organism; they are opportunities for actions (Stoffregen, 2003). Affordances depart from existing understandings of value and meaning since they posit their own definition of both concepts. Perceiving something as an affordance is not perceiving value-free physical objects which are then imbued with meaning; it is perceiving a value-rich ecological object (Sanders, 1993). Affordances are important to adaptive evolution, and any change in one elicits a change in the other: both evolve together. Since affordances are reflections of purposiveness, adaptive goal-driven behaviours of organisms serve to structure and condition affordances themselves. This forms the basis of evolution.

6.6 Co-Evolution: Form and Affordance

The relationship between form and affordance is different to the relationship between form and environment in Modern Synthesis. Affordances are not detached from the properties of form itself. Affordances do not have their own internal dynamics in the same way as the environment. The relationship between an organism and its affordances is reciprocally constituting and reciprocally effecting (they co-evolve/co-variation). In actively responding to an affordance, an organism creates new affordances, and as biological form changes, so too do the affordances. The relationship between an organism and its affordances is "dialectical". They cannot exist independently, and they gain their properties from their relationships with one another, and both properties evolve as a result of this (Levins and Lewontin, 1985). Adaptive evolution is not form-solving adaptive problems posed by the organism's environment, but it is rather creating and responding to an ever-changing system of affordances (the plasticity of development). Adaption is a symbiotic dialectic; affordance is something that is co-created by organisms and their contexts, both of which co-evolve through the dialectic.

6.7 Situated Adaptationism

Situated Adaptationism (Walsh, 2012) does not posit a causal decoupling of organisms and the conditions to which they are adapted. Therefore, it does not rely on explanatory externalism. For this approach, the process of creating novel phenotypes is also the process of creating novel affordances. Adaption, normally construed, means an implicit decoupling of an organism and its environment. To make the metaphor of adaptation work, environments or ecological niches must exist before the organisms in them, otherwise environments cannot *cause* organisms to fill those niches. The history of life is the history of the coming into being of new forms that fit more closely into these pre-existing niches (Lewontin, 2001b). But this raises a dilemma. If we view evolution as adaptation, we see the "autonomous existence of environments independent of living creatures" (Lewontin, 2001b). But if the metaphor of adaptation is abandoned, we cannot explain the role of organisms and their

environments (Lewontin, 2001b). However, "environments of organisms are made by organisms themselves because of their own life activities" (Lewontin, 2001b).

While adaptedness is assessed in terms of suitability to conditions of continual existence, those conditions of existence are largely constituted by the organism. This is known as "construction" (Odling-Smee et al., 2003). There are two forms of construction: causal, and constitutive. In causal construction, organisms cause changes to the physical features of the external environment by their activities. These physical changes in the conditions then affect the organisms. However, this is still an aspect of decoupling. The Churchillian niche (Godfrey-Smith, 2001b) construction argument then posits that niches are not causally autonomous of the organism, but are *explanatorily autonomous*. The environment selects the organisms which are most adept at living in it.

Another interpretation is that organisms are commingled with their niches; there is no separation. Organisms and their niches are a single interacting system: dialectic. *The former version (Churchillian) describes a situation of reciprocal causation, whereas the dialectic describes a situation of reciprocal constitution.* This is *Marxian* dialectical materialism. This dialectic can unfold in the following way: An affordance is what an organism is able to use, and this depends on an organism's capacities, which depends on its context. This can explain a new approach to adaptive evolution. Therefore, it is not a passive form shaped by an autonomous or living environment (the position of Modern Synthesis); organisms have a reciprocally constitutive relationship with their conditions. An organism can constitute its niche by changing the environmental physical parameters (which then also affect the organism). An organism can also make changes to its own form (or make itself more or less sensitive to its conditions) without affecting the environment, which then alters affordances.

6.8 Adaptation Without Adaptationism

The processes occurring "within" organisms—inheritance and development—are explanatorily relevant only insofar as biological form *is not* adaptive. While inheritance and development are conservative and "constrain" form in various ways, selection is "creative" (Mayr, 1976). Therefore, there are two explanations: (1) "externalist" explanations of the adaptedness of form; and (2) "internalist" explanations of the constraints on form. Lewens (2009) argues that the commonality of form between related lineages is explained by the conservativeness of "internal" development, while the differences in form within a lineage are explained by the external "forces of selection". The very idea that there are internalist and externalist explanations rests on the traditional error of supposing that the influence of the environment on form and the effects of inheritance and development can be differentiated and apportioned.

6.9 Internalism and Externalism

Internalist explanations speak to the inner processes of inheritance and development; externalist explanations speak to the notion of the selective influence of the environment in traditional doxa. Amundson (2001) highlighted how this is homologous to the "functional" (Lewens, 2009)[9] versus "structural" debate (as the cause of form), which has long been at the centre of biology (Amundson, 2001). Adaptationists are on the side of functionalism, while developmentalists are on the side of

[9] Functionalism posits that function comes before form and objects have the forms; they do so in order to serve these functions. Structuralism, on the other hand, posits that form or structure comes before function and hence function does not explain form. Form instead is to be understood as autonomous formal/structural properties and processes.

structuralists (Webster and Goodwin, 1996). Situated Darwinism, however, posits that the division between function and structure and form and development is misguided. This also holds true for the inner and outer forces dichotomy. This is because development and inheritance are not inner processes; they are distributed throughout the organism/environmental system. Functions which promote influences on form, should not be understood as being strictly external to the organism itself. Functions are distributed, as mentioned (Amundson, 2005; Walsh, 2015). There is also no division of explanatory labor between processes which conserve form and those which promote adaptive change. Activities of organisms and their ability to exploit and alter affordances explain both conservation and change (Walsh, 2015). Adaptation is thus not the opposition of inner forces (conservative) opposing outer ones (change). Instead, adaptation is a process where organisms by their plasticity jointly mould both organism and affordances by affecting changes in either or both. It is a commingled relationship which does not rely on these dichotomies.

6.10 Adaptation or design?

If evolutionary adaptation is not the process by which biological form is molded to solve the pre-existing problems posed by the environment, then the concept of a biological adaptation bears no particular resemblance to the concept of design. A design *is* a solution to a pre-existing problem; a biological adaptation is not (Griffiths and Gray, 1994). The Modern Synthesis conception of an adaptation as a solution to a design problem is pervasive. It derives its intuitive appeal from the analogy between organisms and machines. Adaptationist versions of the argument from design have substantially the same character. Organisms are cast as “survival machines” built to do their replicators’ bidding (Dawkins, 1982).

Organisms are the “order-from-disorder” entities which are self-building, self-nourishing, self-regulating, homeostatic and goal directed. Hence machine/mechanistic-based analogies/metaphors or description of organisms are ill-suited to their task. Organisms and machines are dissimilar to each other. The mechanistic explanation underplays the impact of adaptive evolution and the distinctiveness of organisms. This also obscures the causes of adaptative evolution and provides an incorrect theory of evolutionary change. Adaptation is not design because it involves *active niche constitution* (for this reason organisms are not designed). Organisms are agents which construct their worlds and their place in it (Walsh, 2015).

6.11 Conclusion

The process of adaptive evolution describes population changes as consequences of organisms responding to and constructing affordances for themselves. Affordances are emergent properties of embedded purposive systems unlike environments. A system which is responsive to affordances is one that can produce changes in its own state or to its environment because these changes are conducive to goal attainment. Seeking goal attainment explains the contribution of those changes. Therefore, affordance implies that certain features of evolution happen because organisms create them in their pursuits. For this to work, there needs to be an account of “purposiveness” and teleology.

According to Modern Synthesis, evolution is “chance caught on the wing”. The paramount source of evolution is random genetic mutation, and natural selection is the sole evolutionary process responsible for the introduction of biases. Therefore, processes that introduce new variants must be unbiased—processes creating evolutionary novelties cannot present an adaptive bias. On the other

hand, Situated Darwinism asserts that evolutionary change is driven by the pursuit of organisms' purposes (given their affordances). In doing so, organisms respond adaptively, which may introduce new, stable phenotypes that are preserved and recur in a population through development. Many of these novelties arise because, given the circumstances, they are beneficial to the survival of the organism. Introduction of evolutionary novelties is therefore biased in favour of the purpose of organisms. Evidence suggests that the adaptiveness of organisms is a precondition for adaptive evolution. Rather than being based on chance, adaptive evolution forms part of an organism's purposive activities.

7 Part G

Ethics is *symbolic*. In anthropology, the first conceptions of ethics and concepts like fairness can be traced back to hunter gatherer times (Tomasello, 2018). Briefly, the earliest evidence of morality came around 400,000 years ago with the hunter gatherers. It arose because:

- Animals demonstrate what happens in the absence of collaboration: a scramble for domination and an overall net loss of food.
- Joint/collective intentionality meant that early humans needed to swap scavenging already eaten or disease-ridden carcases (dangerous) with hunting. Hunting is easier in groups.
- From this collaboration, an objective morality arose as that which enabled the achievement of the common goal. From this objective morality arose the distinction between "we" and "me" (as a secondary personal morality). In other words, the collective and the individual.
- The objective morality was about the common goal and achieving it, the secondary morality was about fairness. From this, other symbolics grew and so did cultures. That is where right and wrong comes from—what is good for the culturally selected common goals.
- Fairness was a notion about sharing after achievement—usually linked to cutting the meat in equal proportions. It was about *rewarding agents of secondary morality*.
- Intentionality was understood to be a means of evolutionary elimination. If you did not contribute or keep up your end of the bargain, then you did not share in the spoils. It was not about an internal state—it was the *performance of an obligation* between agents of the secondary notion of morality in lieu of meeting a common goal.
- As groups grew, some split off, and therefore it was necessary to distinguish one group from another. Thus, group identity was created as a means of *identifying who an agent of secondary morality is within different groups*. This meant that distinctions were necessary, which I suggest would find their origin in the contextual environment of the group (us versus them). This also required indoctrination of infants in these norms and values in order to ensure continuity.

In urban societies, there is much more uncertainty and stress because of the nature of urban arrangements: there is much more anonymity and regular encounters with strangers (Sapolsky, 2017). In urban environments, there is a greater need for there to be a mechanism for enforcing norms among strangers, and people who come and go (Sapolsky, 2017). Therefore, there is a greater need for third party mechanisms where punishment is left to third party institutions like the police, law,

and courts. The key is the uncertainty element. Given all the movement, we attribute cognition to the individual who claims it, and if there is a dispute the law steps in as the arbiter trying to solve the uncertainty. In the context of law, intentionality was not something which subjects judged, but rather it was something for an objective third party to determine (an Other). It was an attribution of "intent" (as an explanation for actions) in circumstances to explain or punish or reward actions. These are third person views—not first or second.

8 References

Abrams, M. H. (1953). *The Mirror and the Lamp: Romantic Theory and the Critical Tradition*. Oxford: Oxford University Press.

Amundson, R. (2001). "Adaptation and development: On the lack of common ground," in *Adaptationism and Optimality*, eds. S. H. Orzack and E. Sober (Cambridge: Cambridge University Press), 303-334.

Amundson, R. (2005). *The Changing Role of the Embryo in Evolution*. Cambridge: Cambridge University Press.

Ayala, F. (1970). Teleological explanations in evolutionary biology. *Philosophy of Science*, 37 (1), 1-15.

Brunnander, B. (2007). What is natural selection? *Biology and Philosophy*, 22 (2), 231-246. doi: 10.1007/s10539-005-9008-4

Carroll, S. B., Grenier, J. K., and Weatherbee, S. D. (2000). *From DNA to Diversity: Molecular Genetics and the Evolution of Animal Design*. Oxford: Wiley-Blackwell.

Chemero, A. (2003). An outline of a theory of affordances. *Ecological Psychology*, 15 (2), 181-195. doi: 10.1207/S15326969ECO1502_5

Chenciner, A. (2012). Poincaré and the three-body problem. *Séminaire Poincaré XVI*, 45-133.

Dawkins, R. (1982). *The Extended Phenotype: The Gene as the Unit of Selection*. Oxford: W. H. Freeman and Company.

Gerhard, J. M., and Kirschner, M. (2007). The theory of facilitated variation. *PNAS*, 104, 8582-8589. doi: 10.1073/pnas.0701035104

Gibson, J. J. (1979). *The Ecological Approach to Visual Perception*. New York: Taylor & Francis.

Godfrey-Smith, P. (1996). *Complexity and the Function of Mind in Nature*. Cambridge: Cambridge University Press.

Godfrey-Smith, P. (2001a). "Three kinds of Adaptationism," in *Adaptationism and Optimality*, eds. S. H. Orzack and E. Sober (Cambridge: Cambridge University Press), 335-357.

Godfrey-Smith, P. (2001b). "Organism, environment and dialectics," in *Thinking About Evolution*, eds. R. Singh, C. Krimbas, D. Paul, et al. (Cambridge: Cambridge University Press), 253-266.

Goertzel, T. (n.d.). The myth of the bell curve. https://crab.rutgers.edu/users/goertzel/normalcurve.htm [Accessed January 23, 2023].

Griffiths, P. E., and Gray, R. D. (1994). Developmental systems and evolutionary explanation. *Journal of Philosophy*, 91, 277-304.

Hall, B. K. (2012). Evolutionary developmental biology (evo-devo): Past, present, and future. *Evo. Edu. Outreach*, 5, 184-193. doi: 10.1007/s12052-012-0418-x

Haugeland, J. (1998). *Having Thought: Essays in the Metaphysics of Mind*. Cambridge: Harvard University Press.

Hawkins, J., and Dawkins, R. (2021). *A Thousand Brains: A New Theory of Intelligence*. New York: Basic Books.

Hendrikse, J. L., Parsons, T. E., and Hallgrímsson, B. (2007). Evolvability as the proper focus of evolutionary developmental biology. *Evolution and Development*, 9 (4), 393-401. doi: 10.1111/j.1525-142X.2007.00176.x

Hidalgo, C. (2021). *How Humans Judge Machines*. Cambridge: MIT Press.

Kant, I., and Bernard, J.H. (ed). (1790). *Critique of Judgment*. New York, NY: Barnes & Noble.

Kant, I. (1998). *Critique of Pure Reason*, translated by P. Guyer and A. Wood. Cambridge: Cambridge University Press (originally published 1781).

Kauffman, S. (1993). *The Origins of Order: Self-Organization and Selection in Evolution*. Oxford: Oxford University Press.

Keil, F.C. (2006). Explanation and understanding. *Annu. Rev. Psychol.*, 57, 227-254. doi: 10.1146/annurev.psych.57.102904.190100

Kirschner, M., and Gerhard, J. (2005). *The Plausibility of Life: Resolving Darwin's Dilemma*. New Haven: Yale University Press.

Kitano, H. (2004). Biological robustness. *Nat. Rev. Genet.*, 5, 826-837. doi: 10.1038/nrg1471

Levin, M. (2022). Technological approach to mind everywhere: An experimentally-grounded framework for understanding diverse bodies and minds. *Front. Syst. Neurosci.*, 16, 1-43. doi: 10.3389/fnsys.2022.768201

Levins, R., and Lewontin, R. C. (1985). *The Dialectical Biologist*. Cambridge: Harvard University Press.

Lewens, T. (2009). Seven types of adaptationism. *Biol. Philos.*, 24, 161-182. doi: 10.1007/s10539-008-9145-7

Lewontin, R. C. (1978). Adaptation. *Scientific American*, 239 (3), 212-231.

Lewontin, R. C. (2001a). *The Triple Helix: Genes, Organisms and Environments*. Cambridge: Harvard University Press.

Lewontin, R. C. (2001b). "Gene, organism and environment," in *Cycles of Contingency: Developmental Systems and Evolution*, eds. S. Oyama, E. P. Griffiths, and R. Gray (Cambridge: MIT Press), 59-66.

Marx, K. (1867). *Das Kapital: Kritik der Politischen Oekonomie*. Hamburg: Verlag von Otto Meissner.

Maynard Smith, J., Burian, R., Kauffman, S., Alberch, P., Campbell, J., Goodwin, B., Lande, R., Raup, D., and Wolpert, L. (1985). Developmental constraints and evolution: A perspective from the Mountain Lake Conference on development and evolution. *Quarterly Review of Biology*, 60 (3), 265-287.

Mayr, E. (1976). *Towards a New Philosophy of Biology*. Cambridge: Harvard University Press.

Mill, J.S. (1843). *A System of Logic, Ratiocinative and Inductive*. London: Savill and Edwards.

Moczek, A. P., Sultan, S., Foster, S., Ledon-Rettig, C., Dworkin, I., Nijhout, H. F., Abouheif, E., and Pfennig, D. W. (2011). The role of developmental plasticity in evolutionary innovation. *Proceedings of the Royal Society*, 2705-2713. doi: 10.1098/rspb.2011.0971

Monod, J. (1971). *Chance and Necessity: An Essay on the Metaphysics of Life*. New York: Vintage Books.

Müller, G. B. (2007). Evo-devo: Extending the evolutionary synthesis. *Nat. Rev. Genet.*, 8, 943-949. doi: 10.1038/nrg2219

Müller, G. B., and Newman, S. A. (2005). The innovation triad: An evodevo agenda. *J. Exp. Zool. (Mol. Dev. Evol.)*, 304B, 487-503. doi: 10.1002/jez.b.21081

Mullin, T. (ed) (1993). *The Nature of Chaos*. Oxford: Clarendon Press.
Noble, D. (2013). Evolution beyond Neo-Darwinism. *J. Exp. Biol.*, 218, 7-13. doi: 10.1242/jeb.106310
Odling-Smee, F. J., Laland, K. N., and Feldman, M. W. (2003). *Niche Construction: The Neglected Process in Evolution*. Princeton: Princeton University Press.
Ott, W. (2020). *Foucher/Desgabets: Translations from the Cartesian Debate on Ideas and Representation*.
Pfennig, D. W., Wund, M. A., Snell-Rood, E. C., Cruickshank, T., Schlichting, C. D., and Moczek, A. P. (2010). Phenotypic plasticity's impacts on diversification and speciation. *Trends in Ecology and Evolution*, 25, 459-467. doi: 10.1016/j.tree.2010.05.006
Raff, R. (1996). *The Shape of Life: Genes, Development and the Evolution of Animal Form*. Chicago: University of Chicago Press.
Sanders, J. T. (1993). Merleau-Ponty, Gibson, and the materiality of meaning. *Man and World*, 26 (3), 287-302. doi: 10.1007/bf01273397
Sapolsky, R. M. (2017). *Behave: The Biology of Humans at our Best and Worst*. New York: Penguin Books.
Schuessler, R. (2019). Probability in Medieval and Renaissance Philosophy. *Stanford Encyclopedia of Philosophy*. https://plato.stanford.edu/entries/probability-medieval-renaissance/ [Accessed January 24, 2023].
Schwenk, K., and Wagner, G. (2004). "The relativism of constraints on phenotypic evolution," in *The Evolution of Complex Phenotypes*, eds. M. Pigliucci, and K. Preston (Oxford: Oxford University Press), 390-408.
Shapiro, J. A. (2011). *Evolution: A View From the 21st Century Perspective*. New Jersey: FT Press.
Shiller, R. J. (2003). From efficient markets theory to behavioral finance. *J Economic Perspectives*, 17(1), 83-104. doi: 10.1257/089533003321164967.
Sommerhoff, G. (1950). *Systems Biology*. Oxford: Oxford University Press.
Stoffregen, T. A. (2003). Affordances as properties of the animal–environment system. *Ecological Psychology*, 15, 115-134. doi: 10.1207/S15326969ECO1502_2
Taleb, N. N. (2007). *The Black Swan: The Impact of the Highly Improbable*. New York: Penguin Books.
Tomasello, M. (2018). The origins of human morality. *Scientific American.* https://www.scientificamerican.com/article/the-origins-of-human-morality/ [Accessed September 25, 2022].
Waddington, C. H. (1957). *The Strategy of the Genes*. London: Routledge.
Wagner, A. (2011). *The Origin of Evolutionary Innovations: A Theory of Transformative Change in Living Systems*. Oxford: Oxford University Press.
Wagner, A. (2012). The role of robustness in phenotypic adaptation and innovation. *Proceedings of the Royal Society*, 279, 1249-1258. doi: 10.1098/rspb.2011.2293
Wagner, A. (2014). *The Arrival of the Fittest: Solving Evolution's Greatest Puzzle*. London: OneWorld.
Wagner, G. P., and Altenberg, L. (1996). Complex adaptations and the evolution of evolvability. *Evolution*, 50, 967-976. doi: 10.2307/2410639
Walsh, D. M. (2012). "Situated Adaptationism," in *The Environment: Philosophy, Science, Ethics*, eds. W. Kabesenche, M. O'Rourke, and M. Slater (Cambridge: MIT Press), 89-116.
Walsh, D. M. (2014). The negotiated organism: Inheritance, development and the method of difference. *Biological Journal of the Linnean Society*, 112 (2), 295-230. doi: 10.1111/bij.12118
Walsh, D.M. (2015). *Organisms, Agency, and Evolution*. Cambridge: Cambridge University Press.

Webster, G., and Goodwin, B. (1996). *Form and Transformation: Generative and Relational Principles in Biology*. Cambridge: Cambridge University Press.
West-Eberhard, M. J. (2003). *Developmental Plasticity and Evolution*. Oxford: Oxford University Press.
Woodward, J. (2000). Explanation and invariance in the special sciences. *Brit. J. Phil. Sci.*, 51, 197-254.
Zhang, J., Conway, J., and Hidalgo, C. A. (2022). Why do people judge humans differently from machines? The role of agency and experience. 1-17. doi: 10.48550/arXiv.2210.10081
